# MMP14 expression levels accurately predict the presence of extranodal extensions in oral squamous cell carcinoma: a retrospective cohort study

**DOI:** 10.1186/s12885-023-10595-x

**Published:** 2023-02-10

**Authors:** Yuri Noda, Mitsuaki Ishida, Ryosuke Yamaka, Yasuhiro Ueno, Tomofumi Sakagami, Takuo Fujisawa, Hiroshi Iwai, Koji Tsuta

**Affiliations:** 1 Department of Pathology and Laboratory Medicine, Kansai Medical University Hospital, 2-3-1 Shin-machi, 573-1191 Hirakata, Osaka Japan; 2 Department of Pathology, Kansai Medical University, 2-5-1 Shin-machi, 573- 1010 Hirakata, Osaka Japan; 3 Department of Pathology, Osaka Medical and Pharmaceutical University, 2-7 Daigaku-Machi, 569-8686 Takatsuki, Osaka Japan; 4 Department of Radiology, Kansai Medical University Hospital, 2-3-1 Shinmachi, 573-1191 Hirakata, Osaka Japan; 5 Department of Otolaryngology, Head and Neck Surgery, Kansai Medical University Hospital, 2-3-1 Shinmachi, 573-1191 Hirakata, Osaka Japan

**Keywords:** Extranodal extension, Oral squamous cell carcinoma, Matrix metalloproteinase 14, Membrane-type 1 matrix metalloproteinase, Cancer-associated fibroblast, Predictor

## Abstract

**Background:**

Extranodal extension (ENE) is an adverse prognostic factor for oral squamous cell carcinoma (OSCC), and patients with OSCC along with ENE require neck dissection. In this study, we developed a novel ENE histology-based pathological predictor using MMP14 expression patterns in small biopsy specimens.

**Methods:**

A total of 71 surgically resected tissue, 64 dissected lymph node (LN), and 46 biopsy specimens were collected from 71 patients with OSCC. Immunohistochemical analyses of total MMP14 expression in the tumour nest and cancer-associated fibroblasts (CAFs) were performed using the MMP14 co-scoring system (high- or low-risk). The association analysis of MMP14 expression in metastatic LNs was performed with respect to the presence and absence of ENE. Clinicopathological analyses and multivariate examinations were performed to assess the risks of metastasis and ENE presence. The predictive value of ENE and the impact of ENE and MMP14 expression on 5-year overall survival were examined.

**Results:**

High-risk MMP14 expression was detected in metastatic LN specimens with ENE. MMP14 expression in tumour nests and CAFs and its overexpression at the tumour–stromal interface significantly correlated with the presence of ENE. The MMP14 co-scoring system was an independent risk predictor for ENE, with sensitivity, specificity, and accuracy of over 80% in biopsy samples; patients with a high risk in the MMP14 co-scoring system had significantly worse prognoses in both resections and biopsies.

**Conclusion:**

The MMP14 co-scoring system accurately predicted ENE presence and poor prognosis via immunohistochemical evaluation of small biopsies. This system is a simple, accurate, and inexpensive immunohistochemical approach that can be used in routine pathological diagnosis for effective treatment planning.

**Supplementary Information:**

The online version contains supplementary material available at 10.1186/s12885-023-10595-x.

## Background

An extranodal extension (ENE) is an extension of tumour cells through the lymph node (LN) capsule into the surrounding connective tissue [1, 2]. It is the most important prognostic factor in human papillomavirus-positive and -negative head and neck squamous cell carcinoma (HNSCC) and is associated with increased locoregional recurrence, distant metastasis, and decreased survival [3–6]. Patients with pathological ENE (pENE) need high-dose chemoradiotherapy [7]; however, this is only diagnosed by post-operative pathological examination. Clinical and radiological examinations do not satisfactorily evaluate ENE presence (ENE+), and accuracy ranges from 7.0 to 85.0% [8–10].

Oral squamous cell carcinoma (OSCC) is a type of HNSCC [1, 7]. For patients with OSCC, with even minor ENE (< 2.0 mm), the 5-year overall survival is poorer than that of patients without ENE (30.4% vs. 63.1%) [11], and 40% of them experience occult LN metastasis [8–10]. Therefore, OSCC is more strongly recommended for neck dissection than other HNSCCs, even if there are no clinically observed LN metastases [12, 13]. However, neck dissection may negatively affect host immunity and tumour response to immune checkpoint inhibitors [14] as well as incur post-operative cosmetic and functional problems. Thus, an accurate pre-operative prediction method is needed for identifying patients at high risk of ENE+—those who truly need cervical dissection.

Some histological predictive features of ENE + include tumour budding (TB), tumour-infiltrating lymphocytes (TILs), and desmoplastic reaction (DR) [15–22]. TB, TILs, and DRs in the tumour microenvironment (TME) at the tumour–stromal interface (TSI) in primary OSCC reflect tumour TME remodelling ability and are highly concordant with TB, TILs, and DRs at the ENE site [15]. However, histological predictive methods are not useful for biopsies with little or no TSI, wherein the accuracy remains below 80% [15].

Matrix metalloproteinases (MMPs), especially MMP2, 3, 9 and 14, derived from cancer-associated fibroblasts (CAFs) and tumour cells, are powerful TME remodelling factors, which enhance tumour invasiveness and metastasis in OSCC [16, 20, 22–24]. MMP2, 3, and 9 are expressed within the tumour cell cytoplasm, MMP2 and 9 are gelatinases [25, 26], while MMP3 is a stromelysin [26]. MMP14 is a membrane-type-1 MMP expressed at the tumour membrane and cleaves gelatine, fibronectin, and laminin and regulates invadopodium development [27, 28].

Through this study, we aimed to develop a histology-based ENE + prediction method for application to small pre-operative biopsy specimens. Our newly developed MMP14 scoring system is a novel ENE + prediction method with more accuracy than those for TB, TILs, and DRs, and is applicable to biopsies with over 80% accuracy.

## Methods

### Patients and case selection

This retrospective study included 71 patients with OSCC who underwent surgical resection between 2011 and 2020 (Additional File 1).

### Histopathological evaluations

The histological assessments conducted and definitions of TSI, CAFs, TB [16–19], TILs [20, 21], DR [22], and clinicopathological features such ENE are presented in Additional File 2. Histological assessments of TB [16–19], TILs [20, 21], DR [22] and CAFs at the TSI and clinicopathological evaluations were performed based on haematoxylin and eosin (H&E) staining of biopsies, resected primary sites, and LNs from patients with OSCC.

### Immunohistochemistry and immunohistochemical scoring of resections, biopsies, and LN dissections

**Fig. 1:**
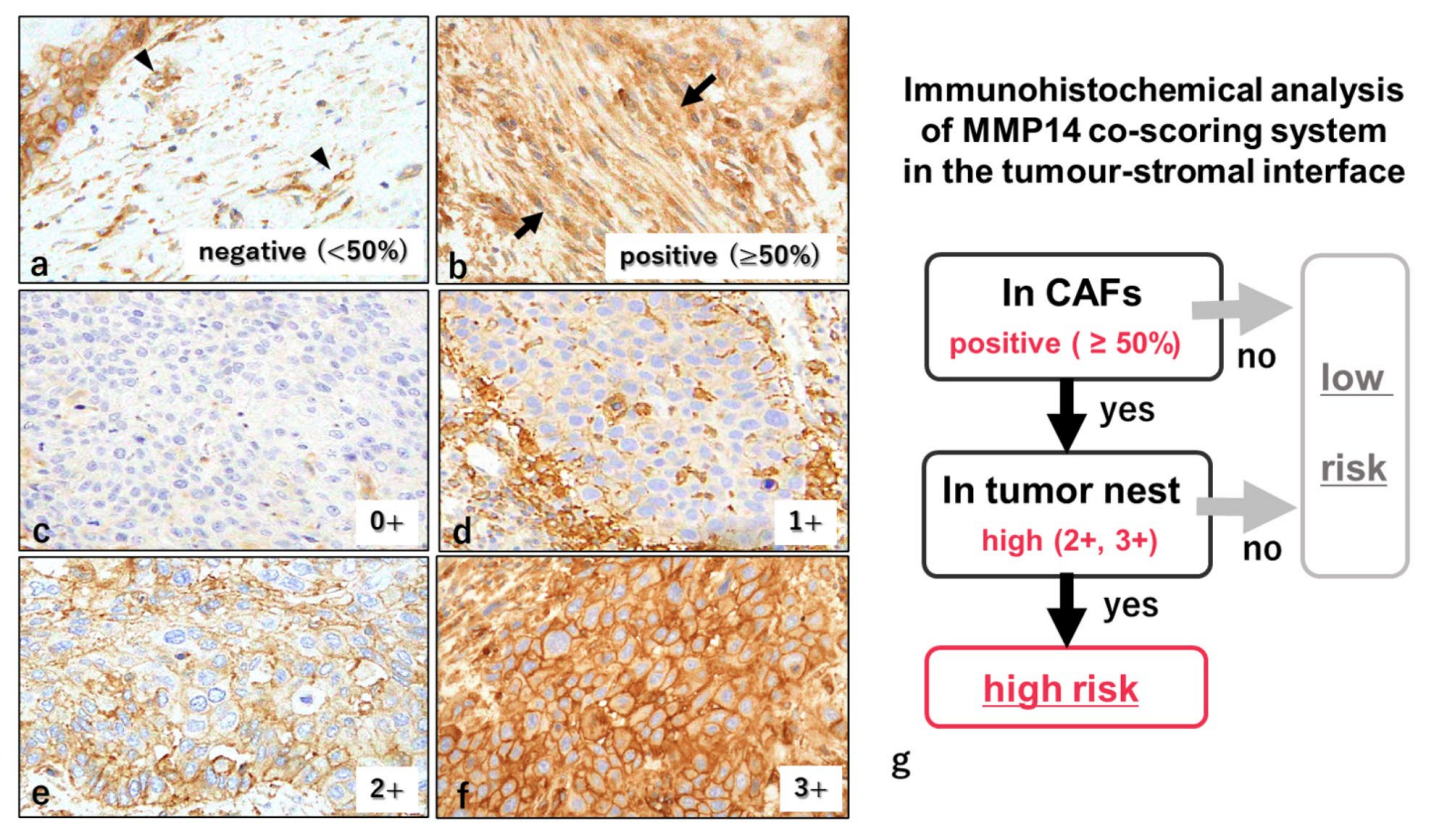
Immunohistochemical analysis of MMP14 expression in tumour nests and CAFs at the primary TSI. Immunohistochemical staining of MMP14 of OSCC resected specimens in TSI. MMP14 expression in CAFs: negative, CAFs < 50% (**a**); positive, CAFs ≥ 50% (**b**). MMP14 expression at the tumour nest in the TSI (**c−g**). MMP14 score at the tumour nest: 0, none (**c**); 1, weak cytoplasmic expression without membrane expression (**d**); 2, moderate cytoplasmic expression or incomplete membrane expression (**e**); and 3, strong cytoplasmic expression or complete membrane expression (**f**). Assessment of MMP14 established ranges from 0 to 3+; sample scoring of 2 + to 3 + was ‘high’ and 0 to 1 + was ‘low’. Flowchart of the MMP14 co-scoring system (**g**). Total MMP14 expression in tumours and CAFs was examined as follows: high-risk (cases that were CAFs-positive and whose tumour scores were high) and low-risk (cases that were not high-risk). Original magnification: **a−f**, 400× HPF. CAFs, cancer-associated fibroblast; TSI, tumour–stromal interface

Immunohistochemical staining of MMPs and evaluation of MMP expression in tumour biopsy, surgically resected specimens, and intranodal metastatic areas and in the ENE area in dissected LNs were performed. The evaluation methods for MMP2, 3, 9, and 14 expression are described in Additional Files 3 and 4. The MMP14 expression evaluation method is shown in Fig. 1.

### Statistical analysis

This information is provided in Additional File 5.

## Results

### Clinicopathological features of 71 matched resections and 46 biopsy specimens from patients with OSCC

In our study, we included 71 surgically resected tissue, 64 matched-neck dissection, and 46 matched biopsy specimens from 71 patients with OSCC (Table 1).

**Table 1:** Clinicopathological features of matched 71 resection and 46 biopsy specimens from patients with OSCC

	All patients (n = 71)		Patients with biopsies (n = 46)	
**Characteristics**	No. of patients	%	No. of patients	%
**Age**	Range = 39–91		Range = 39–91	
	(median = 69, mean = 67.8)		(median = 68.5, mean = 67.5)	
> 65	24	34%	18	39%
≤ 65	47	66%	28	61%
**Sex**				
Male	43	61%	26	57%
Female	28	39%	20	43%
**Location**				
Buccal mucosa	8	11%	4	9%
Gingiva	15	21%	7	15%
Tongue	48	68%	35	76%
**pT**				
1,2	17	24%	14	30%
3,4	54	76%	32	70%
**LN metastasis**				
Absent	26	37%	23	50%
Present	45 (27 cases were ENE+)	63%	23 (10 cases were ENE+)	50%
**pN**				
0,1	38	54%	32	70%
2,3	33	46%	14	30%

OSCC, oral squamous cell carcinoma; ENE, extranodal extension; pT, pathological T; pN, pathological N; LN, lymph node; ENE+, presence of extranodal extension

LN dissection was performed for 64 patients, LN metastasis (pLN+) was observed for 45, 27 cases displayed ENE (pLN+/pENE+), and 18 did not exhibit pENE (pLN+/pENE-). A pLN+/pENE- case was excluded from the analyses owing to the poor FFPE quality; 44 pLN+/pENE cases were available for immunohistochemical examination. Of the 28 cases without LN metastasis (pLN-), LN dissection was conducted in 21 cases and not in the remaining 7 cases, where clinical LN metastasis had not yet occurred. For the 71 cases, 46 matched biopsy specimens were available for analysis, as summarised in Table 1. Of these, 23, 10, and 23 cases were pLN+, pLN+/pENE+, and pLN-/ENE-, respectively.

### Comparison of MMP expression at the dissected metastatic LN sites with and without ENE and MMP expression at the ENE site

**Fig. 2:**
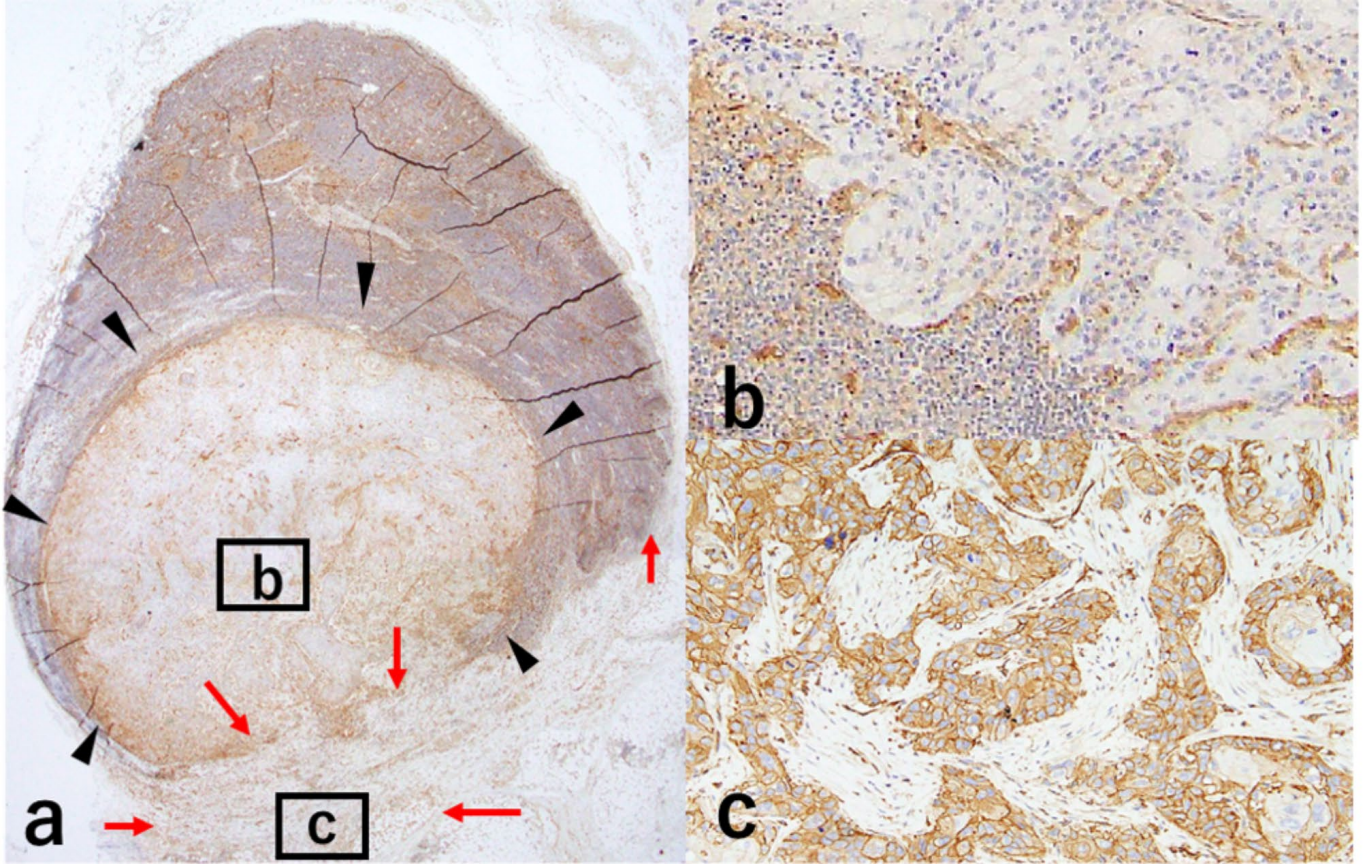
Immunohistochemical analysis of MMP14 expression in the tumour nest and CAFs at the metastatic LN. MMP14 expression in LNs with ENE (**a−c**). At the ENE site, MMP14 expression in the tumour nest is higher than that in the intranodal tumour nest (**a−c**: black arrowheads show cancer nest inside the LN; red arrows show cancer nest at the ENE site). Intranodal tumour nest score was 0+; there was no CAFs (**b**). At the ENE site, the tumour nest had a score of 3+; CAFs was observed; and MMP14 was expressed in CAFs (**c**). Original magnification: a, 5× high power field (HPF); b and c, 20× HPF. ENE, extranodal extension; CAFs, cancer-associated fibroblasts; LN, lymph node

**Fig. 3:**
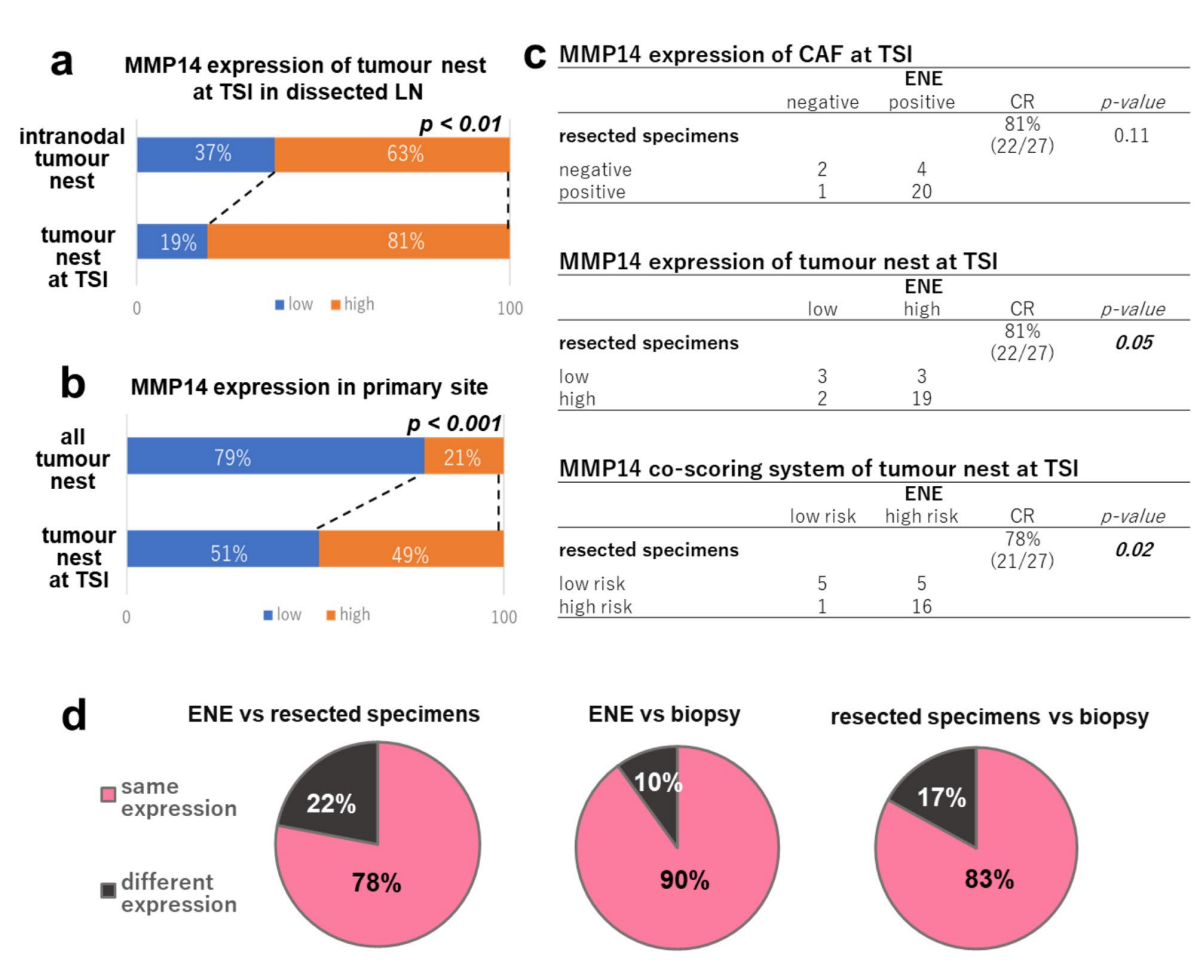
MMP14 expression at the TSI in the primary site and dissected LNs. High MMP14 expression in tumour nest is more at the ENE site than at the intranodal site (a, 63% vs. 81%). High MMP14 expression in the tumour nest is more at the TSI than at the tumour nest in the primary site (b, 21% vs. 49%). The high CR of MMP14 expression at the TSI is detected in CAFs (c, ENE vs. resected specimens, 81%, *p* = 0.11), tumour nest (c, 81%, *p* = 0.05), and co-scoring system (c and d, left, 78%, *p* = 0.02). The CR of MMP14 co-scoring expression between the ENE and biopsy (d, middle, 90%) and the resected specimens and biopsy (d, right, 83%). CAFs, cancer-associated fibroblasts; CR, concordance rate; ENE, extranodal extension; LN, lymph node; TSI, tumour–stromal interface

MMP expression data are described in Additional File 6. MMP2, 3, 9, and 14 expression in the tumour nest and CAFs was more profound at the ENE site than in the intranodal component (*p* < 0.01; Figs. 2 and 3a and Additional File 7). In the 44 cases of LN metastasis, there was no association between MMP2, 3, and 9 expression and ENE+/- (all *p > 0.05*, Table 2). Only MMP14 expression in CAFs and the tumour nest was associated with ENE+ (all *p < 0.05*). High-risk cases according to the MMP14 co-scoring system, including the total MMP14 enzymatic activity derived from tumour nest and CAFs, were associated with ENE+; 83% of them had ENE+ (*p < 0.05*).

**Table 2:** Association between MMP expression profiles and ENE presence in metastatic LNs

	LN(+)/ENE(-)	LN(+)/ENE(+)	*p-*value
**Intranodal MMP14 expression**			
**CAFs**			***< 0.01***
Negative	11 (65%)	6 (35%)	
Positive	6 (22%)	21 (78%)	
**Tumour nest**			***0.03***
Low	12 (55%)	10 (45%)	
High	5 (23%)	17 (77%)	
**MMP14 co-scoring system**			***0.01***
Low-risk	14 (54%)	12 (46%)	
High-risk	3 (17%)	15 (83%)	
**Intranodal MMP2 expression**			
**CAFs**			0.98
Negative	7 (39%)	11 (61%)	
Positive	10 (38%)	16 (62%)	
**Tumour nest**			0.51
Low	9 (35%)	17 (65%)	
High	8 (44%)	10 (56%)	
**Intranodal MMP3 expression**			
**CAFs**			0.8
Negative	12 (38%)	20 (63%)	
Positive	5 (42%)	7 (58%)	
**Tumour nest**			0.58
Low	13 (39%)	20 (61%)	
High	4 (36%)	7 (64%)	
**Intranodal MMP9 expression**			
**CAFs**			0.49
Negative	10 (43%)	13 (57%)	
Positive	7 (33%)	14 (67%)	
**Tumour nest**			0.5
Low	15 (38%)	25 (63%)	
High	2 (50%)	2 (50%)	

LN, lymph node; LN+, presence of lymph node metastasis; ENE, extranodal extension; ENE-, absence of extranodal extension; ENE+, presence of extranodal extension; CAFs, cancer-associated fibroblasts. Boldface indicates statistically significant values

### Association of MMP expression at the TSI between the resected specimens and the ENE site of metastatic LN specimens

High MMP2, 3, 9, and 14 expression in the tumour nest was more predominant at the TSI than in the tumour nest in primary resected specimens (*p* < 0.001, Fig. 3b; Additional File 7). The concordance rate (CR) was highest for MMP14 expression, compared to that of other MMPs (CAFs, 81%, *p* = 0.11; tumour nest, 81%, *p* = 0.05; co-expression system, 78%, *p* < 0.01) (Fig. 3c, d, and Additional File 6).

### Association of clinicopathological features with MMP14 and other MMPs at the TSI of primary tumour specimens

We determined if the expression pattern of MMPs in the primary resected specimens was associated with pENE presence (pENE+) and TME activity-related features (TB, DR, and TILs). The clinicopathological features of all 71 patients with OSCC are compiled in Additional File 6.

MMP14 expression in CAFs and the tumour nest at the TSI were significantly associated with pENE + and TME features, such as TB, pLN+, pN2/3, lymphatic invasion (Ly), and perineural invasion (Pn) (*p* < 0.05, Table 3). Additionally, MMP14 expression in CAFs was associated with pT, pDOI > 10 mm, invasion pattern, DR, TILs, and V (*p* < 0.05, Table 3). The high-risk group of the MMP14 co-scoring system was significantly associated with pENE + and TB and also pN+, pN2/3, pT, invasion pattern, Ly, and Pn (*p* < 0.05, Table 3). Age, sex, and locations were not significantly associated with MMP14 expression in the CAFs, tumour nest and co-scoring system (data not shown). Moreover, MMP2 expression in CAFs and MMP9 expression in the tumour nest at the TSI was significantly associated with ENE + but not with TME activity-related features (TB, DR, and TILs) (Additional Files 8 and 9). No associations were found between pENE + and the expression of other MMPs in the tumour nest and CAFs at the TSI (Additional Files 8–10) and the expression of all MMPs in all tumour nests (MMP2, 3, 9; data not shown; MMP14, Additional File 11). Therefore, we considered MMP14 to be relevant.

**Table 3:** Association of clinicopathological features with MMP14 expression in the tumour nest and CAFs of 71 surgically resected OSCC specimens

	MMP14 expression in CAFs at the TSI			MMP14 expression in the tumour nest at the TSI			MMP14 co-scoring system		
	Negative	Positive	*p*	Low	High	*p*	Low- risk	High- risk	*p*
**pT**			***< 0.001***			0.44			***< 0.01***
1.2	16	1		10	7		17	0	
3.4	16	38		26	28		30	24	
**pDOI**			***< 0.001***			0.22			0.22
≤ 10 mm	17	4		13	8		13	8	
> 10 mm	15	35		23	27		23	27	
**Lymph node metastasis**			***< 0.001***			***0.02***			***< 0.001***
(-)	21	5		18	8		25	1	
(+)	11	34		18	27		22	23	
**pN**			***< 0.001***			***0.02***			***< 0.01***
0,1	24	14		24	14		31	7	
2,3	8	25		12	21		16	17	
ENE			***< 0.01***			***< 0.001***			***< 0.001***
(-)	26	18		30	14		37	7	
(+)	6	21		6	21		10	17	
**Differentiation**			0.37			0.37			0.58
Well	17	28		24	21		27	18	
Moderate	13	11		12	12		18	6	
Poor	2	0		0	2		2	0	
**Invasion pattern**			***0.03***			0.06			***0.047***
1.2	6	1		6	1		7	0	
3.4c.4d	26	38		30	34		40	24	
**DR**			***< 0.001***			0.12			0.21
Mature	17	6		22	15		27	10	
Immature	15	33		14	20		20	14	
**TB**			***0.02***			***< 0.01***			***< 0.01***
Low	19	12		23	8		28	3	
High	13	27		13	27		19	21	
**TILs**			***0.02***			0.54			0.08
High	19	12		17	14		24	7	
Low	13	27		19	21		23	17	
**Ly**			***< 0.001***			***0.01***			***0.01***
(-)	15	5		15	5		15	5	
(+)	17	34		21	30		21	30	
**V**			***< 0.001***			0.05			0.06
(-)	16	1		12	5		12	5	
(+)	16	38		24	30		24	30	
**Pn**			***< 0.001***			***0.04***			***0.048***
(-)	16	6		15	7		15	7	
(+)	16	33		21	28		21	28	

CAFs, cancer-associated fibroblasts; OSCC, oral squamous cell carcinoma; TSI, tumour–stromal interface; pT, pathological T; pDOI, pathological depth of invasion; pN, pathological N; DR, depth of invasion; TB, tumour budding; TILs, tumour-infiltrating lymphocytes; Ly, lymphatic invasion; V, vascular invasion; p, p-value; Pn, perineural invasion. Boldface indicates statistically significant values

### Association between MMP14 expression at the TSI in biopsy specimens and the ENE site

**Fig. 4:**
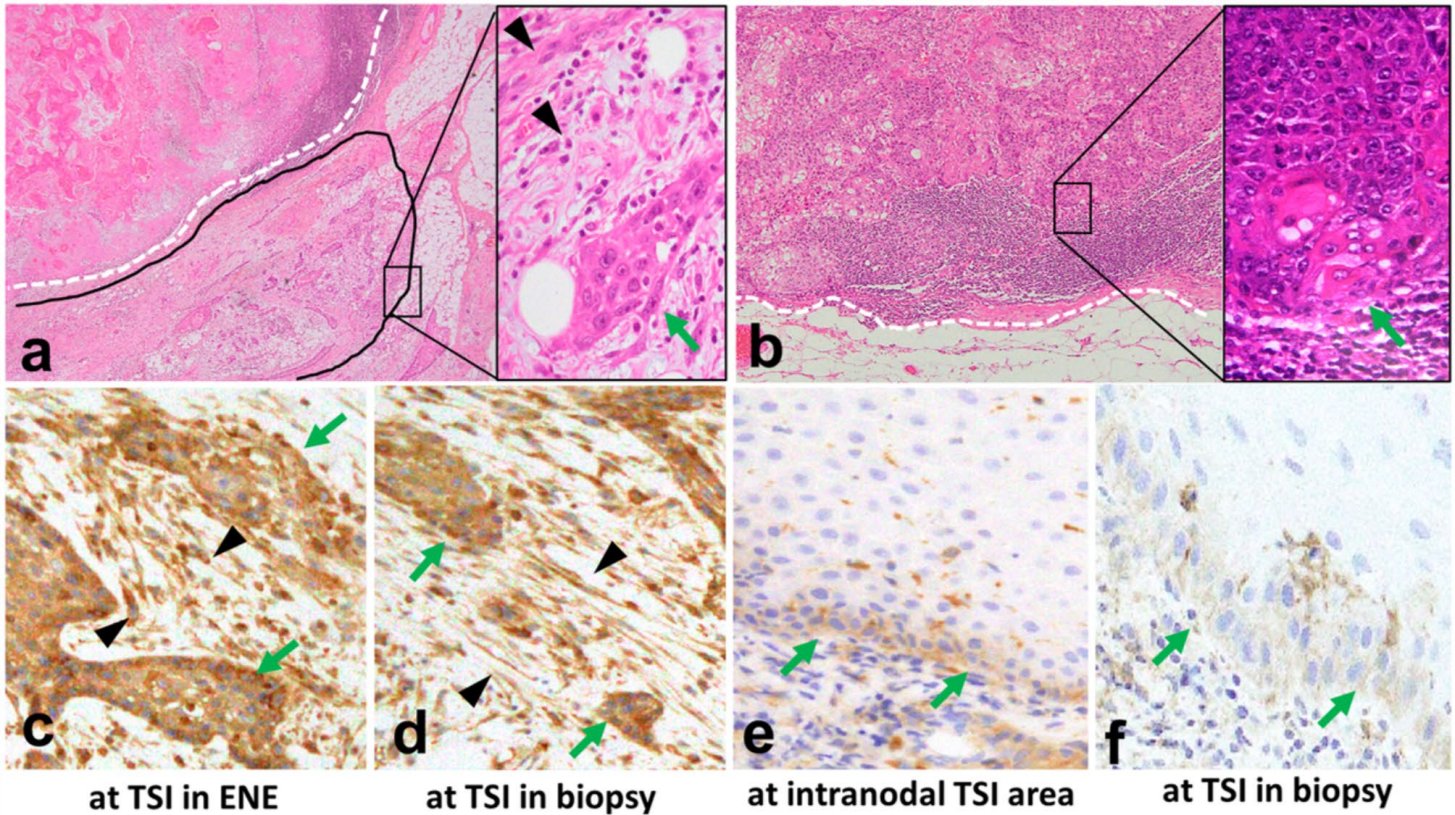
Association between MMP14 at the TSI in biopsy and the ENE site MMP14 expression in the tumour nest (black arrowhead) and CAFs (green arrows) in metastatic LNs with ENE (**a, c**) and without ENE (**b, e**), and the matched biopsy (**d, f**) is consistent. MMP14 expression in the tumour nest (3+, **c** and **d**) and CAFs (positive, **c** and **d**). MMP14 expression in the tumour nest (1+, **e** and **f**) and CAFs (negative, **e** and **f**). Original magnification, **a** and **b**, **left**, 5× high power field (HPF); **a** and **b**, **right**, **c–f**, 40× HPF. TSI, tumour–stromal interface; ENE, extranodal extension; CAFs, cancer-associated fibroblasts; LN, lymph node

At the ENE site, the CR of MMP14 expression in the tumour nest, CAFs, and the risk of the co-scoring system at the TSI in biopsy specimens was 90% (9/10; *p* < 0.05 Figs. 3d and 4a–f, and Additional File 12). Moreover, a high CR of MMP14 expression between the resected and biopsy specimens was found for the tumour nest (65%), CAFs (74%), and co-scoring system (83%; *p* < 0.01, Fig. 3d and Additional File 12).

### Association of clinicopathological features with MMP14 at the TSI in biopsy

Clinicopathological analysis of MMP14 expression in the biopsy samples was performed. CAFs and tumoural MMP14 expression and the MMP14 co-scoring system were significantly associated with pENE+, pN+, pN2/3, and TILs (*p* < 0.05, Table 4). Furthermore, association between MMP14 expression in CAFs and clinical depth of invasion (cDOI) > 10 mm was detected (*p* < 0.05). MMP14 expression in tumour nest and the MMP14 co-scoring system were also associated with DR (*p* < 0.05).

**Table 4:** Association of clinicopathological features with MMP14 expression in the tumour nest and CAFs of 46 OSCC biopsy specimens

	MMP14 expression in CAFs at the TSI (biopsies)			MMP14 expression in the tumour nest at the TSI (biopsies)			MMP14 co-scoring system (biopsies)		
	Negative	Positive	*p*	Low	High	*p*	Low- risk	High- risk	*p*
**pT**			***0.01***			***0.01***			0.05
1.2	12	2		12	2		13	1	
3.4	15	17		15	17		21	11	
**cDOI**			***0.01***			0.17			0.08
≤ 10 mm	14	3		12	5		15	2	
> 10 mm	13	16		15	14		19	10	
**Lymph node metastasis**			***< 0.01***			***< 0.01***			***< 0.001***
(-)	19	4		19	4		23	0	
(+)	8	15		8	15		11	12	
**pN**			***< 0.01***			***0.03***			***< 0.001***
0,1	23	9		22	10		28	4	
2,3	4	10		5	9		6	8	
**ENE**			***< 0.001***			***< 0.01***			***< 0.001***
(-)	26	10		25	11		32	4	
(+)	1	9		2	8		2	8	
**Differentiation**			0.58			0.58			0.58
Well	20	14		14	14		19	9	
Moderate	6	5		11	5		13	3	
Poor	1	0		2	0		2	0	
**Invasion formation**			0.11			0.45			0.28
1.2	4	0		3	1		4	0	
3.4c.4d	23	19		24	18		30	12	
**DR**			0.33			***0.04***			***0.04***
Mature	10	5		12	3		14	1	
Immature	17	14		15	16		20	11	
**TB**			0.24			0.11			0.09
Low	14	7		15	6		18	3	
High	13	12		12	13		16	9	
**TILs**			***0.03***			***0.01***			***0.01***
High	14	4		15	3		17	1	
Low	13	15		12	16		17	11	

CAFs, cancer-associated fibroblasts; OSCC, oral squamous cell carcinoma; TSI, tumour–stromal interface; pT, pathological T; pDOI, pathological depth of invasion; pN, pathological N; DR, depth of invasion; TB, tumour budding; TILs, tumour-infiltrating lymphocytes; Ly, lymphatic invasion; V, vascular invasion; p, p-value; Pn, perineural invasion. Boldface indicates statistically significant values

### Predictive factor for ENE + in resection and biopsy specimens

Bivariate analysis revealed a significant association between ENE + and c/pDOI > 10 mm and between ENE + and MMP14 expression in the tumour nest (high) and CAFs (positive); the analysis was based on the co-scoring system (high risk) in biopsies and resected specimens (all *p* < 0.05, Table 5). However, no association was found between ENE + and TME activity-related histological factors (DR-I, TB-H, and TILs-L).

**Table 5:** Bivariate and multivariate logistic regression analyses of ENE using surgically resected and biopsy specimens

	Bivariate			Multivariate			
	Odds ratio	95% CI	*p-*value	SE	Odds ratio	95% CI	*p-*value
**Resection specimens (n = 71)**							
pT3,4	6.466	1.346–31.058	***0.011***	-	-	-	0.230
pDOI > 10 mm	3.620	1.066–12.299	***0.033***	-	-	-	0.508
Invasion pattern	1.603	0.288–8.906	0.587	-	-	-	-
DR-I	3.046	0.972–9.543	0.050	-	-	-	-
TB-H	3.429	1.205–9.753	0.018	-	-	-	-
TILs-L	1.552	0.583–4.135	0.378	-	-	-	-
MMP14 at the tumour nest (high)	7.500	2.479–22.691	***< 0.001***	-	-	-	0.058
MMP14 in CAFs (positive)	5.056	1.703–15.011	***< 0.01***	-	-	-	0.536
MMP14 co-scoring system (high-risk)	8.986	2.921–27.642	***< 0.001***	0.573	8.986	2.921–27.642	***< 0.001***
**Biopsy specimens (n = 46)**							
cDOI > 10 mm	7.200	0.824–62.937	***0.046***	-	-	-	0.183
DR-I	5.727	0.653–50.258	0.09	-	-	-	-
TB-H	2.000	0.458–8.725	0.35	-	-	-	-
TILs-L	0.250	0.047–1.344	0.09	-	-	-	-
MMP14 at the tumour nest (high)	9.091	1.654–49.965	***< 0.01***	-	-	-	0.458
MMP14 in CAFs (positive)	23.400	2.626-209.279	***< 0.001***	-	-	-	0.289
MMP14 co-scoring system (high-risk)	32.0000	4.953-206.762	***< 0.001***	0.952	32.000	4.953-206.761	***< 0.001***

CI, confidence interval; SE, standard error; pT, pathological T; pDOI, pathological depth of invasion; DR-I, immature desmoplastic reaction; TB-H, high tumour budding; TILs-L, low-grade tumour-infiltrating lymphocytes; CAFs, tumour-associated fibroblasts; cDOI, clinical depth of invasion. Boldface indicates statistically significant values

Multivariate logistic regression analysis revealed that the MMP14 co-scoring system was an independent factor of ENE + both in resected (odds ratio [OR] = 8.986, 95% confidence interval [CI] = 2.921−27.642; *p* < 0.001) and biopsy specimens (OR = 32.0, 95% CI = 4.953−206.761; *p* < 0.001).

### Predictive value of MMP14 expression for ENE + risk factors

In the resected specimens, the MMP14 co-scoring system (high risk) showed high specificity (sensitivity, 63%; specificity, 84%; positive predictive value [PPV], 71%; NPV, 79%; accuracy, 76%; AUC = 0.735; 95% CI, 0.628–0.843; Table 6). In the biopsy specimens, the MMP14 co-scoring system (high risk) showed a high predictive value for ENE+ (sensitivity, 80%; specificity, 89%; PPV, 67%; NPV, 94%; accuracy, 87%; AUC = 0.844; 95% CI, 0.704–0.985). In addition, MMP14 expression in the tumour nest of biopsy specimens showed a high predictive value for predicting ENE+ (sensitivity, 80%; specificity, 72%; PPV, 42%; NPV, 93%; accuracy, 72%; AUC = 0.811; 95% CI, 0.688–0.934).

**Table 6:** Performance of MMP14 expression as a predictor of extranodal extension

MMP14 expression	Sensitivity	Specificity	PPN	NPV	Accuracy	AUC	95% CI	*p-*value	Cohort proportion (no.; yes:no)
Surgically resected specimens									
CAFs (positive)	78%	59%	46%	81%	66%	0.684	0.576–0.793	*< 0.001*	(39:32)
Tumour nest (high)	78%	68%	60%	83%	72%	0.730	0.6238–0.8358	*< 0.01*	(35:36)
Co-scoring system (high risk)	63%	84%	71%	79%	76%	0.735	0.628–0.843	*< 0.01*	(24:47)
Biopsy specimens									
CAFs (positive)	90%	72%	47%	96%	76%	0.811	0.688–0.934	*< 0.001*	(19:27)
tumour nest (high)	80%	72%	42%	93%	72%	0.747	0.596–0.899	*0.01*	(19:27)
Co-scoring system (high-risk)	80%	89%	67%	94%	87%	0.844	0.704–0.985	*< 0.01*	(12:34)

PPV, positive predictive value; NPV, negative predictive value; AUC, area under the roc curve; CI, confidence intervals; CAFs, cancer-associated fibroblasts. Boldface indicates statistically significant values

### Five-year overall survival of patients with OSCC who underwent 71 resections and 46 biopsies in whom ENE was absent or present and for whom MMP14 co-scoring system indicated high or low risk

**Fig. 5:**
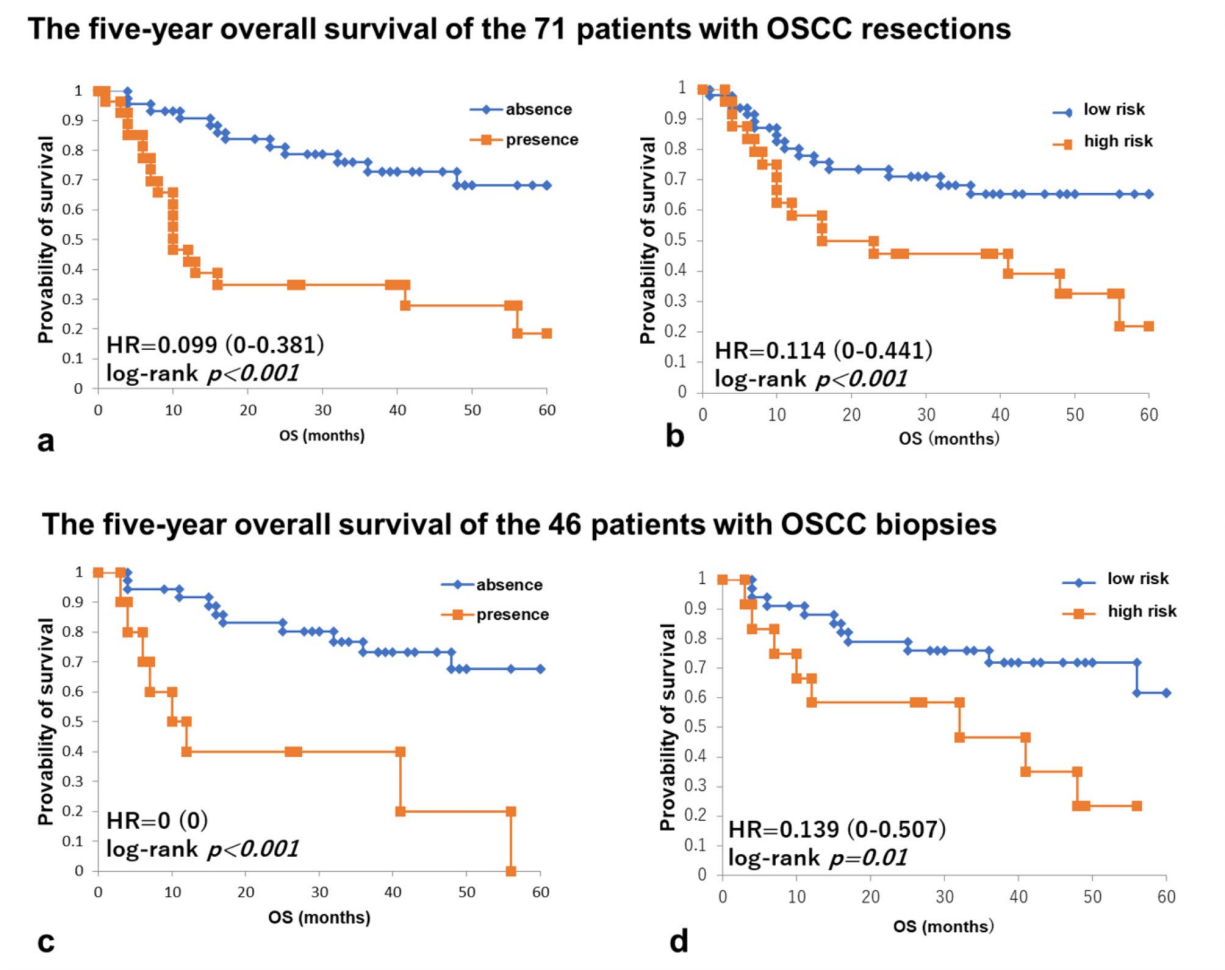
Five-year overall survival of patients with OSCC who underwent 71 resections and 46 biopsies in whom ENE was absent or present and for whom the MMP14 co-scoring system indicated high risk or low risk In 71 resected patients with or without ENE **(a)** and at high risk or low risk according to the MMP14 co-scoring system **(b)**. In 46 patients also evaluated by biopsies with or without ENE **(c)** and at high risk or low risk according to the MMP14 co-scoring system **(d).** Notably, patients with ENE and at high-risk according to the MMP14 co-scoring system had significantly worse prognosis in both resection and biopsies

In 71 patients with surgically resected OSCC, patients with ENE had a significantly worse overall survival (OS) compared to patients without ENE (HR, 0.099, CI: 0–0.381, *p < 0.001*, Fig. 5a). Moreover, among the 71 patients for whom MMP14 co-scoring system was evaluated at the TSI in resection, the high-risk patients had a significantly worse OS compared to the low-risk patients (HR 0.114, CI: 0–0.441, *p = 0.01*, Fig. 5b).

In 46 patients for whom biopsies were also evaluated, patients with ENE had a significantly worse OS than patients without ENE (HR, 0, CI: 0–0, *p < 0.001*, Fig. 5c). Furthermore, among the 46 patients for whom the MMP14 co-scoring system was evaluated at the TSI in biopsies, the high-risk patients had a significantly worse OS than the low-risk patients (HR, 0.139, CI: 0–0.507, *p = 0.01*, Fig. 5d).

## Discussion

This study indicated that MMP14 expression in metastatic LNs is associated with ENE + and that MMP14 levels in tumours and CAFs at the TSI are highly concordant with the ENE sites. In biopsies and resections, associations of MMP14 expression level with ENE + and TME remodelling features were detected, along with clinicopathological metastasis and invasive features. High risk was determined by the MMP14 co-scoring system as an independent factor for ENE + and a poor prognosis factor for 5-year OS in both biopsy and resected specimens; notably, the system was highly accurate for detecting ENE + in biopsies. Moreover, MMP14 expression in tumour nests was a reliable predictor in small specimens without connective tissues. Immunohistochemical analysis of MMP14 expression level is simple, easy, and useful in assessing the ENE risk of patients with OSCC without any clinical data. Thus, the developed method can be used in routine pathological diagnoses for planning pre-operative treatment.

MMP14 overexpression in tumour nests and CAFs was more often observed at metastatic LNs with ENE than in those without ENE and at the TSI in the primary and ENE sites; the expression at these sites was similar, even in biopsies. These results indicate that MMP14 expression in tumour nests and CAFs is associated with ENE development. MMP14 localises at the surface-membrane expression in OSCC, particularly in the invasive area, and promotes extracellular matrix degradation [28–31]. In addition, MMP14 derived from tumour cells activates mesenchymal cells and CAFs in the TME [32–36]. CAF-derived MMP14 releases the matrix, promoting OSCC invasion [28–31, 37]. As enzymes and tumour cells demonstrate the most activity at the TSI [19, 22–24], it is reasonable to assume that ENE develops significantly in LNs involved in OSCC metastasis, wherein MMP14 is overexpressed. To date, no studies have assessed the expression of MMPs at the TSI and ENE sites nor the immunohistochemical expression of membranous MMP14 [38]. Considering the high CR of MMP14 at the TSI at primary and ENE sites, the MMP14 co-scoring system could predict tumour invasiveness at the ENE site by assessing it at the primary TSI.

MMP14 expression in tumour nests and CAFs in primary TSI showed a significant association with clinicopathological pENE+, TME activity-related features (TB, DR, and TILs), and invasiveness and metastatic features. MMP2 and MMP9 were also associated with pENE + but did not show any association with TME activity-related features. MMP2, 3, 9, and 14, derived from CAFs and tumour cells in OSCC, are important enzymes for metastasis [38–42]; however, their role in ENE development has not been examined. In vivo studies have revealed that MMP14 in the TME creates a suitable primary and pre-metastatic niche in the LN for tumour survival during epithelial–mesenchymal transition (EMT) [32–36], which may be attributable for the similar expression profiles of MMP14 at TSI and ENE sites. In summary, MMP14, particularly at the TSI, is the most predominant enzyme in OSCC for ENE + and metastasis.

MMP14 expression in resected specimens, particularly CAFs, was associated with TME activity and remodelling factors, namely, TB-high, DR-immature, and TILs-low [21, 23]. TB and DR are important features of EMT, which is a fundamental process for cancer metastasis at the TSI [43]. TILs are histological features relating to tumour immunity; TILs-low is associated with the presence of LN metastasis and the pN stage [15]. In combination with previous studies, our present results indicate a positive correlation between MMP14 expression at the TSI and clinicopathological features of metastasis, which may be linked to EMT, and that tumour immunity might affect the metastatic process. Therefore, a co-scoring system based on MMP14 expression in the tumour and CAFs at the TSI comprehensively demonstrates the response of the TME activity. MMP14 expression might be useful for evaluating the ENE + risk and metastatic and invasion ability.

Similar to resected specimens, in biopsies, MMP14 expression in CAFs and tumour nests and its expression based on the co-scoring system revealed high concordance with MMP14 expression at the ENE site. Furthermore, in biopsies, MMP14 expression in tumour nests and CAFs and the co-scoring system were associated with ENE + and TME activity-related features, such as DR and TILs. Supported by high CR, MMP14 expression level might be useful for indicating ENE+, even in biopsies, which indicate fewer TME components than resections.

Multivariate analysis showed that the MMP14 co-scoring system is an independent ENE-predictor for biopsy and resected specimens. Moreover, high-risk cases according to the MMP14 co-scoring system had significantly worse prognosis in both resections and biopsies. Additionally, the system is a novel and accurate method for detecting ENE and determining poor prognoses based on histology alone. This approach showed higher sensitivity and a PPV in biopsy specimens than previously used methods that evaluated DR-H/TILs-L/cDOI > 10 mm (sensitivity 70%, specificity 77%) [15]. The significant association among DR, TILs, and MMP14 co-expression may explain the high sensitivity and specificity of the MMP14 co-scoring system. A previous study that detected ENE based on immunohistochemistry using OSCC resected specimens showed high specificity; however, it required two different antibodies, was not useful for biopsies, and did not evaluate impact as a prognosis marker [44]. Moreover, in one of our approaches, MMP14 expression in tumour nests could be used for ENE prediction in biopsies without stroma; this is an extremely important feature as OSCC biopsy specimens often lack connective tissues, as suggested by the analysis of patient prognoses.

Our study had some limitations. First, the number of included cases was limited; thus, a more detailed examination with a larger sample size including LN metastasis and ENE is needed. Second, the usefulness of the MMP14 co-scoring system employing immunohistochemistry may also be limited in OSCC cases that require demineralisation treatment and patients undergoing pre-operative treatment were not examined. Third, the present study focused on MMP proteins at TSI using immunohistochemistry, which can be evaluated in general hospitals. Further study is needed, including a comprehensive analysis using a large database of genes to clarify the significance of MMP14 in ENE development from biological and clinical perspectives [45–48].

## Conclusion

The MMP14 co-scoring system is a novel ENE-prediction method using biopsy specimens and applies to many OSCC cases. It is highly accurate and can be conducted using a basic, rapid pathological analysis. We strongly believe that our approach is applicable to routine pathological analysis and can be included in patient reports, thereby helping healthcare professionals determine surgical methods and plan pre-operative treatments, such as determining whether patients with OSCC require neck surgery or chemoradiotherapy.

## Electronic supplementary material

Below is the link to the electronic supplementary material.

Supplementary Material 1

Supplementary Material 2

Supplementary Material 3

Supplementary Material 4

Supplementary Material 5

Supplementary Material 6

Supplementary Material 7

Supplementary Material 8

Supplementary Material 9

Supplementary Material 10

Supplementary Material 11

Supplementary Material 12

## Data Availability

All data analysed during this study are included in this published article and its additional files.

## List of abbreviations

AJCC: American Joint Committee on Cancer
AUC: Area under the ROC curve
CAFs: Cancer-associated fibroblasts
cDOI: Clinical depth of invasion
cENE: Clinically defined extranodal extension
CI: Confidence interval
DOI: Depth of invasion
DR: Desmoplastic reaction
DR-I: Immature desmoplastic reaction
DR-M: Mature desmoplastic reaction
EMT: Epithelial–mesenchymal transition, ENE:extranodal extension
H&E: Haematoxylin and eosin
HR: Hazard ratio
HNSCC: Head and neck squamous cell carcinoma
MMP: Matrix metalloproteinases
NPV: Negative predictive value
OR: Odds ratio
OS: Overall survival
OSCC: Oral squamous cell carcinoma
pDOI: Pathological depth of invasion
PPV: Positive predictive value
pT: Pathological T
SCC: Squamous cell carcinoma
SE: Standard error
TB: Tumour budding
TB-H: High tumour budding
TB-L: Low tumour budding
TME: Tumour microenvironment
TILs: Tumour-infiltrating lymphocytes
TILs-L: Low-grade tumour-infiltrating lymphocytes
TILs-H: High-grade tumour-infiltrating lymphocytes
TSI: Tumour–stromal interface.
