# Supplementary material for: MMP14 expression levels accurately predict the presence of extranodal extensions in oral squamous cell carcinoma: a retrospective cohort study
Source: BMC Cancer. 2023 Feb 10;23:142. doi: 10.1186/s12885-023-10595-x (PMC9921360; doi:10.1186/s12885-023-10595-x)
Supplement: Supplementary file 1 — Supplementary Material 1 [file 12885_2023_10595_MOESM1_ESM.docx]

**Additional File 1. Demographics of patients with OSCC selected for the present study**
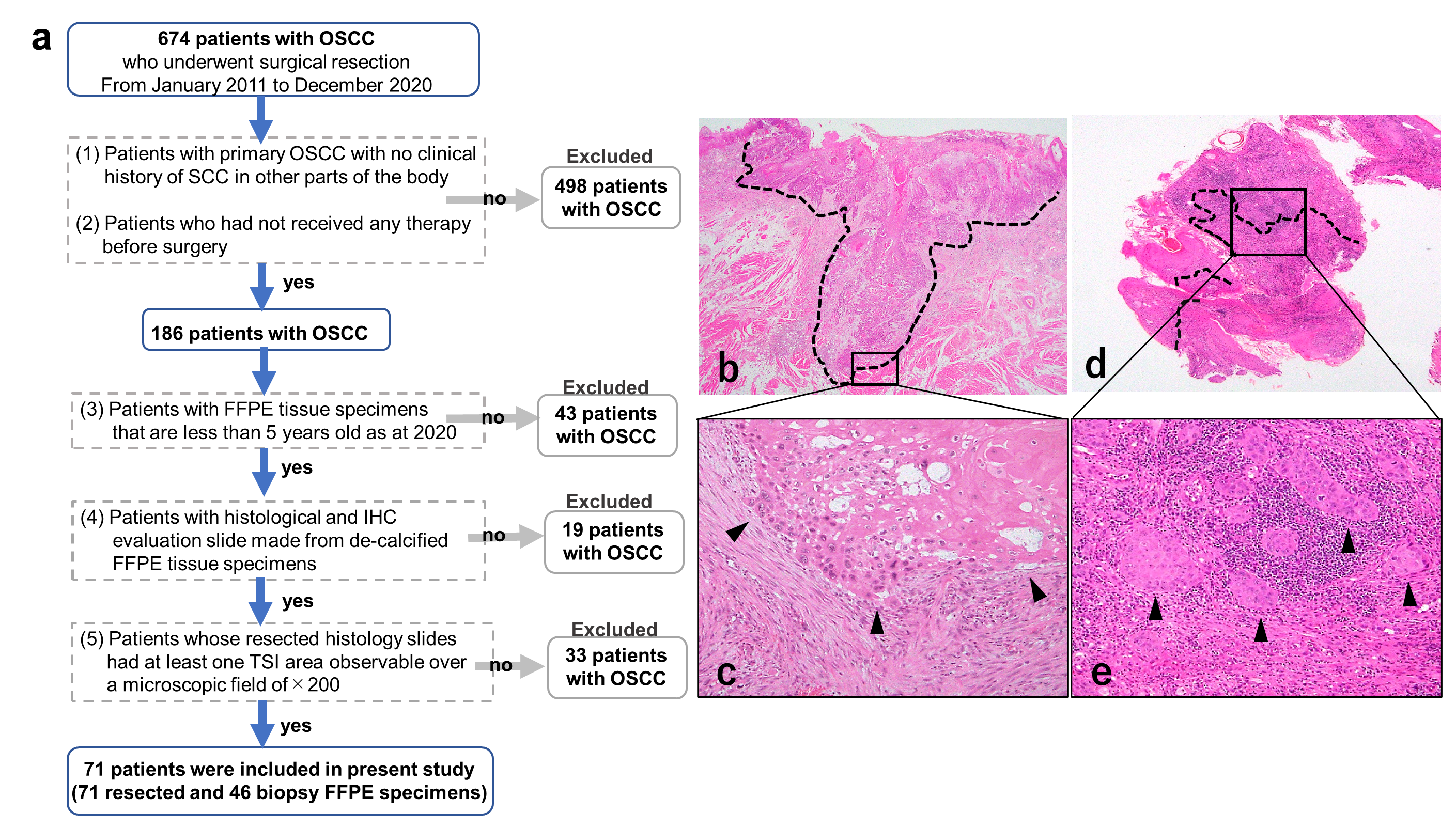


This retrospective study included 674 patients with OSCC who underwent surgical resection at the Department of Otorhinolaryngology, Head and Neck Surgery, Kansai Medical University Hospital, between January 2011 and December 2020. Clinical data were collected from the patient medical health records.

Selection criteria of participants were described in terms of both demographics and clinical/medical factors: (**a**) inclusion and exclusion criteria for 674 patients who had an initial histological diagnosis of OSCC and (1) no clinical history of SCC in other parts of the body, (2) had not received any therapy before surgery, (3) whose biopsy and resected formalin-fixed, paraffin-embedded (FFPE) tissue specimens were less than 5 years old as of 2020, (4) whose histological and immunohistochemical evaluation slides were made from non-decalcified FFPE tissue specimens, and (5) whose resected histology slides had at least one TSI area observable over a microscopic field of 200×. (**b, c**) Resected histology slides and (**d, e**) biopsy show a TSI area including each invasive tumour nest and TME containing cancer-associated fibroblasts and lymphocytes. The black line indicates TSI; black arrowheads indicate tumour nest. Based on these parameters, 603 patients were excluded, and 71 patients with OSCC were finally evaluated.

This study was conducted following the principles of the Declaration of Helsinki and was approved by the Institutional Review Board of the Kansai Medical University Hospital (Approval＃2020289). Informed consent was obtained from patients using the opt-out methodology, owing to the study's retrospective design with no new risk to the participants. Information regarding inclusion criteria and the opportunity to opt out is provided on the hospital’s website (https://www.kmu.ac.jp/hirakata/index.html).

OSCC, oral squamous cell carcinoma; SCC, squamous cell carcinoma; FFPE, formalin-fixed, paraffin-embedded; TME, tumour microenvironment; TSI, tumour–stromal interface; IHC, immunohistochemistry. Original magnification: 10× (b), 200× (c, e), 40× (d).
