# Supplementary material for: MMP14 expression levels accurately predict the presence of extranodal extensions in oral squamous cell carcinoma: a retrospective cohort study
Source: BMC Cancer. 2023 Feb 10;23:142. doi: 10.1186/s12885-023-10595-x (PMC9921360; doi:10.1186/s12885-023-10595-x)
Supplement: Supplementary file 2 — Supplementary Material 2 [file 12885_2023_10595_MOESM2_ESM.docx]

**Additional File 2. Histological assessment of the 71 patients with OSCC, based on haematoxylin and eosin (H&E) staining**

Histopathological evaluations were performed based on H&E staining of samples collected from 71 patients with OSCC diagnosed according to the World Health Organization grading system [1] and the 8^th^ edition of the AJCC staging manual [2] by an oral pathologist (YN) blinded to patient clinical information.

Histological assessments of the tumour budding (TB), desmoplastic reaction (DR), and tumour-infiltrating lymphocytes (TILs) were performed using biopsy, surgically resected primary site, and dissected lymph node (LN) specimens. The assessment of tissue specimens was performed as previously described [3]. The tumour–stromal interface (TSI) is defined as the tumour microenvironment (TME) area around the tumour invasion front, including the tumour nest and the tumour-associated stroma within a 200× microscopic field of the selected biopsy, resected primary site, and dissected LN specimens (Additional File 1). TB is characterised by small tumour nests composed of < 5 cells at the TSI [4-7]. TILs exhibit highly specific immunological reactivity against the tumour cells [8,9]. DR consists of keloidal collagen or myxoid stroma, the formation of which is induced by cancer-associated fibroblasts (CAFs) [10]. DRs, TILs, and DB in each slide were recorded based on the appearance of the highest activity features at the TSI: DR-immature (DR-I), TB-high (TB-H), and TIL-low (TIL-L) were considered to be histological features of high TME remodelling activity [3,11]. In addition, evaluation of other clinicopathological factors, such as age, sex, location, tumour progression and invasiveness (pT and pDOI), metastatic features (presence of LN metastasis, N+; progress of LN metastasis, pN2/3), extranodal extention (ENE), and others (differentiation; lymphatic invasion, Ly+; vascular invasion, V+; and perineural invasion, Pn+), was performed as previously described [3]. Furthermore, CAFs was defined as > 10 stromal cells arranged in band-like proliferation, showing spindle-shaped and plump nuclei at the TSI when observed at 20× magnification under a microscope.

Histological analysis of dissected LNs was performed as follows: metastatic LN with ENE presence (LN+/ENE+) was evaluated based on the observed LN with the largest ENE diameter, and clinicopathological assessment of ENE (c/pENE) was performed as previously [3]. Additionally, metastatic LN without ENE (LN+/ENE-) and LN without metastasis (LN-) were evaluated based on the observed LN with the largest diameter. Histological evaluation of DR, TIL, and TB in dissected metastatic LNs was performed using the TSI in each intranodal area and extranodal ENE area.

**References**

1. El-Naggar AK, Chan JKC. WHO classification of head and neck tumours. 4th ed. Grandis JR, Takata T, Grandis J, Slootweg PJ, editors. Lyon: IARC; 2017.

2. Ridge JA, Lydiatt WM, Patel SG. Lip and oral cavity. In: Amin MB, Edge S, Greene F, editors. AJCC cancer staging manual. 8th ed. New York: Springer; 2017. p. 79–94.

3. Noda Y, Ishida M, Ueno Y, Fujisawa T, Iwai H, Tsuta K. Novel pathological predictive factors for extranodal extension in oral squamous cell carcinoma: a retrospective cohort study based on tumor budding, desmoplastic reaction, tumor-infiltrating lymphocytes, and depth of invasion. BMC Cancer 2022;22:402.

4. Hase K, Shatney C, Johnson D, Trollope M, Vierra M. Prognostic value of tumor ‘budding’ in patients with colorectal cancer. Dis Colon Rectum 1993;36:627–35.

5. Lugli A, Kirsch R, Ajioka Y, Bosman F, Cathomas G, Dawson H, et al. Recommendations for reporting tumor budding in colorectal cancer based on the International Tumor Budding Consensus Conference (ITBCC) 2016. Mod Pathol 2017;30:1299–311.

6. Almangush A, Pirinen M, Heikkinen I, Mäkitie AA, Salo T, Leivo I. Tumour budding in oral squamous cell carcinoma: a meta-analysis. Br J Cancer 2018;118:577–86.

7. Grigore AD, Jolly MK, Jia D, Farach-Carson MC, Levine H. Tumor budding: the name is EMT. Partial EMT. J Clin Med 2016;5:51.

8. Heikkinen I, Bello IO, Wahab A, Hagström J, Haglund C, Coletta RD, et al. Assessment of tumor-infiltrating lymphocytes predicts the behavior of early-stage oral tongue cancer. Am J Surg Pathol 2019;43:1392–96.

9. Ueno H, Kanemitsu Y, Sekine S, Ishiguro M, Ito E, Hashiguchi Y, et al. Desmoplastic pattern at the tumor front defines poor-prognosis subtypes of colorectal cancer. Am J Surg Pathol 2017;41:1506–12.

10. Bryne M. Is the invasive front of an oral carcinoma the most important area for prognostication? Oral Dis 1998;4:70–7.

11．Chiu K, Hosni A, Huang SH, Tong L, Xu W, Lu L, et al. The potential impact and usability of the eighth edition TNM staging classification in oral cavity cancer. Clin Oncol (R Coll Radiol) 2021;33:e442–9.
