# Supplementary material for: MMP14 expression levels accurately predict the presence of extranodal extensions in oral squamous cell carcinoma: a retrospective cohort study
Source: BMC Cancer. 2023 Feb 10;23:142. doi: 10.1186/s12885-023-10595-x (PMC9921360; doi:10.1186/s12885-023-10595-x)
Supplement: Supplementary file 3 — Supplementary Material 3 [file 12885_2023_10595_MOESM3_ESM.docx]

**Additional File 3. Immunohistochemistry and immunohistochemical scoring of resections, biopsies, and lymph node (LN) dissections**

Immunohistochemical analysis was performed using the BOND III fully automated stainer (Leica Biosystems, Melbourne, Australia), following the manufacturer’s protocols. The slides were visualised with BOND™ Polymer Refine Detection (Leica Biosystems, Melbourne, Australia). Primary antibodies against MMP2 (clone **17B11, Novocastra**, Newcastle upon Tyne, UK; dilution 1:500), MMP3 (clone EP1186Y, Abcam, MA, USA, dilution 1:100), MMP9 (clone EP1254, Abcam, dilution 1:500), and MMP14 (clone **EP1264Y,** Abcam, dilution 1:500) were used.

MMP expression in tumour biopsies, surgically resected specimens, and intranodal metastatic areas and in the extranodal extension area in dissected LN was evaluated. Forty-six biopsy specimens were collected from the primary tumor site. MMP distribution was scored based on the tumour nest at the tumour–stromal interface (TSI), all tumour nests in a selected specimen, and the cancer-associated fibroblasts (CAFs) at the TSI. The present study did not evaluate MMP expression in CAFs within the whole tumour in a selected specimen because performing an accurate assessment and comparison of MMP expression in CAFs between tumour nests was challenging. MMP expression was considered positive if it showed the same or stronger expression level as the vascular wall or smooth muscle that was the endogenous positive control.

MMP expression in CAFs at the TSI was evaluated as follows: negative (< 50% of CAFs cytoplasmic staining) or positive (≥ 50% of CAFs cytoplasmic staining) (Additional file 4).

The expression of MMP2, 3, and 9 in the tumour nest was evaluated as follows: low (< 50 % of tumour cytoplasmic staining) or high (≥ 50% of tumour cytoplasmic staining) (Additional File 4) [1-4].

As MMP14 was expressed strongly on the membrane, the evaluation was performed using the 2015 United Kingdom-recommended guidelines for HER2 immunohistochemical assessment [5]. This was performed for all tumour nests and the TSI. The assessment of MMP14 expression establishes ranges from 0 to 3+. However, in the present study, sample scorings of 2+ to 3+ were regarded as ‘high’, and those from 0 to 1+ were considered ‘low’. Furthermore, the total MMP14 expression in tumours and CAFs was examined as follows: high-risk (cases that were CAF-positive and whose tumour scores were high) and low-risk (cases that were not high-risk). This approach was named the ‘MMP14 co-scoring system’.

The expression of MMP2, 3, 9, and 14 was considered to be increasing when the expression level of MMPs changed from ‘low’ to ‘high’.

Slides were scored by two pathologists (YN and MI) using a dual-headed microscope; the scoring was performed in a manner blinded to patient clinical information.

**References**

1. Hase K, Shatney C, Johnson D, Trollope M, Vierra M. Prognostic value of tumor ‘budding’ in patients with colorectal cancer. Dis Colon Rectum 1993;36:627-35.

2. Heikkinen I, Bello IO, Wahab A, Hagström J, Haglund C, Coletta RD, et al. Assessment of tumor-infiltrating lymphocytes predicts the behavior of early-stage oral tongue cancer. Am J Surg Pathol 2019;43:1392-6.

3. Bryne M. Is the invasive front of an oral carcinoma the most important area for prognostication? Oral Dis 1998;4:70-7.

4. Mughees M, SenGupta A, Khowal S, Wajid S. Mechanism of tumour microenvironment in the progression and development of oral cancer. Mol Biol Rep 2021;48:1773-86.

5. Rakha EA, Pinder SE, Bartlett JM, Ibrahim M, Starczynski J, Carder PJ, et al. Updated UK recommendations for HER2 assessment in breast cancer. J Clin Pathol 2015;68:93-9.
