# Supplementary material for: MMP14 expression levels accurately predict the presence of extranodal extensions in oral squamous cell carcinoma: a retrospective cohort study
Source: BMC Cancer. 2023 Feb 10;23:142. doi: 10.1186/s12885-023-10595-x (PMC9921360; doi:10.1186/s12885-023-10595-x)
Supplement: Supplementary file 4 — Supplementary Material 4 [file 12885_2023_10595_MOESM4_ESM.docx]

**Additional File 4. Immunohistochemical analysis of MMP2, 3, and 9 expression at tumour nests and cancer-associated fibroblasts**


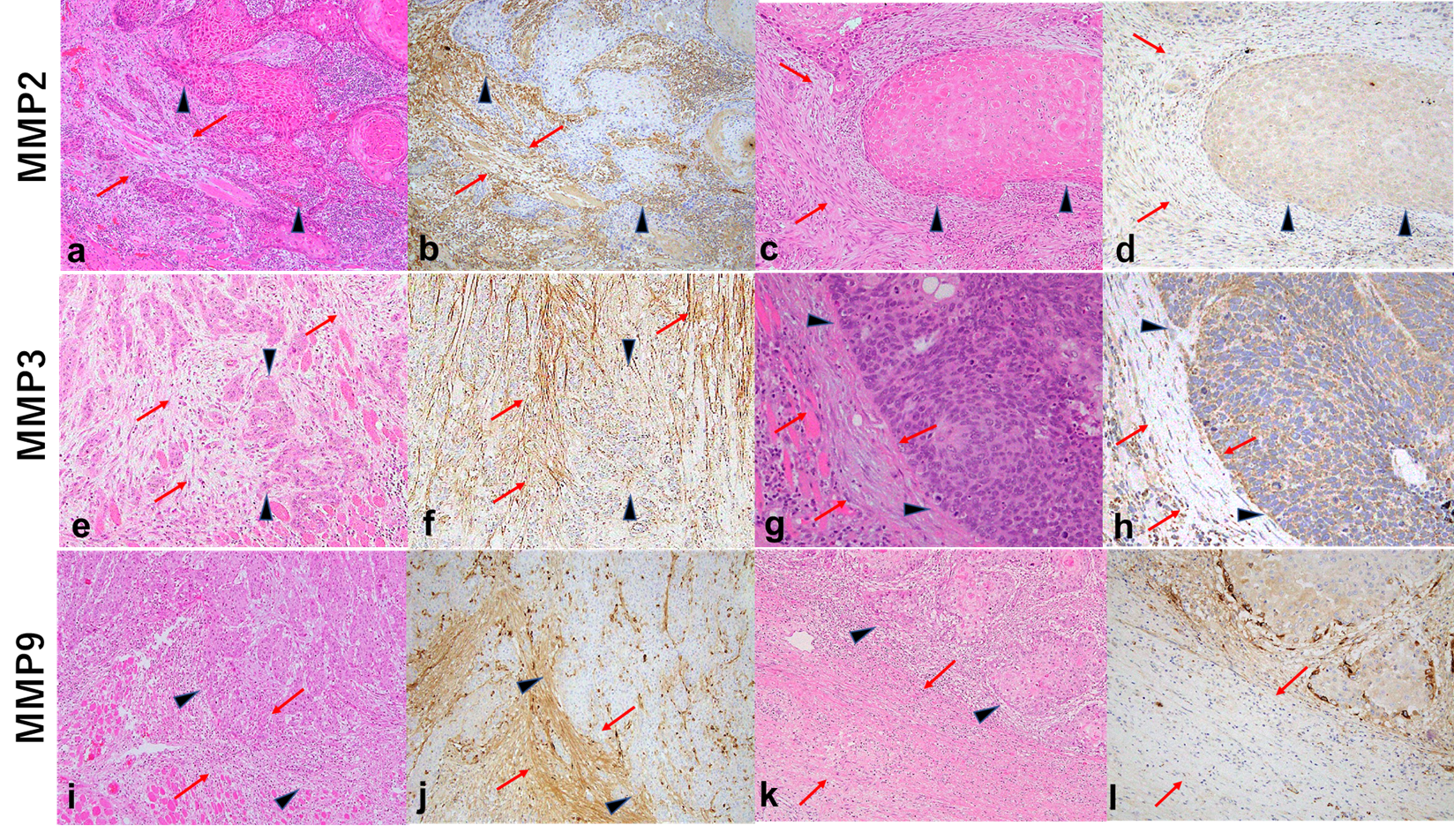


MMP2 expression score in OSCC (**a−d**) tumour nest: ‘low’ (**a**) and ‘high’ (**c**), and cancer-associated fibroblasts (CAFs): ‘positive’ (**b**) and ‘negative’ (**d**). MMP3 expression scores in OSCC (**e−h**) tumour nest: ‘low’ (**e**) and ‘high’ (**g**), and CAFs: ‘positive’ (**f**) and ‘negative’ (**h**). MMP9 expression scores in OSCC (**i−l**) tumour nest: ‘low’ (**i**) and ‘high’ **(k**), and CAFs: ‘positive’ (**j**) and ‘negative’ (**l**).

MMP, matrix metalloproteinase; OSCC, oral squamous cell carcinoma.

Black arrowheads indicate tumour nest; blue arrows indicate CAFs. Original magnification, 200×.
