# Supplementary material for: MMP14 expression levels accurately predict the presence of extranodal extensions in oral squamous cell carcinoma: a retrospective cohort study
Source: BMC Cancer. 2023 Feb 10;23:142. doi: 10.1186/s12885-023-10595-x (PMC9921360; doi:10.1186/s12885-023-10595-x)
Supplement: Supplementary file 5 — Supplementary Material 5 [file 12885_2023_10595_MOESM5_ESM.docx]

**Additional File 5. Statistical analysis**

Correlations between MMP expression were determined using the chi-square or Fisher’s exact test. The Cox proportional hazards model was used to estimate independent factors for the presence of extranodal extension. The diagnostic value of the risk factors was assessed by calculating sensitivity, specificity, positive predictive value (PPV), negative predictive value (NPV), and accuracy. Furthermore, the receiver operating characteristic (ROC) curve and the area under curve (AUC) were used to evaluate and compare the predictive value of predictors. The 5-year overall survival (OS) was evaluated using the Kaplan–Meier method, and differences between survival curves were tested for statistical significance using the log-rank test. Statistical analysis was performed using the SPSS software (v20.0; IBM Corp., Armonk, NY, USA). Significance was set at *p* < 0.05.I
