# Supplementary material for: MMP14 expression levels accurately predict the presence of extranodal extensions in oral squamous cell carcinoma: a retrospective cohort study
Source: BMC Cancer. 2023 Feb 10;23:142. doi: 10.1186/s12885-023-10595-x (PMC9921360; doi:10.1186/s12885-023-10595-x)
Supplement: Supplementary file 7 — Supplementary Material 7 [file 12885_2023_10595_MOESM7_ESM.docx]

**Additional File 7. Expression of MMP2, 3, and 9 and concordance rates of their expression at the ENE site in dissected LNs and at the TSI in resected specimens**


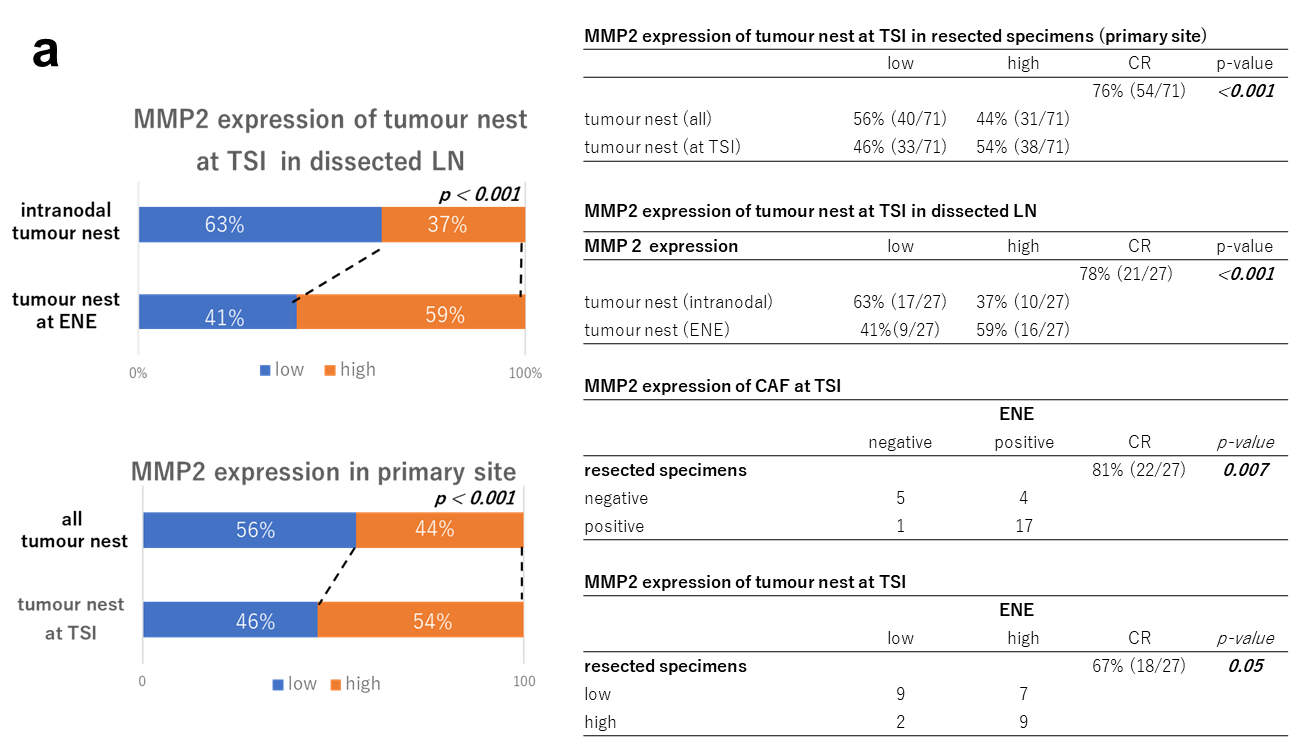


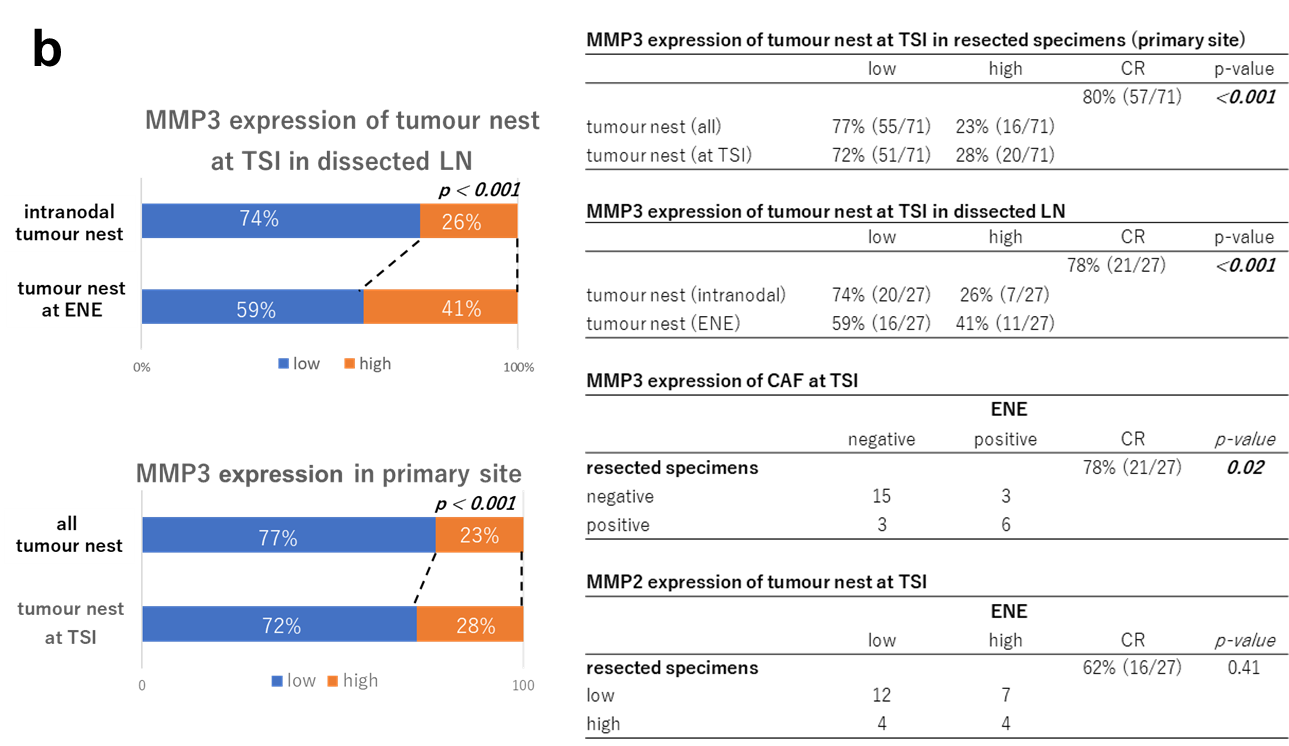


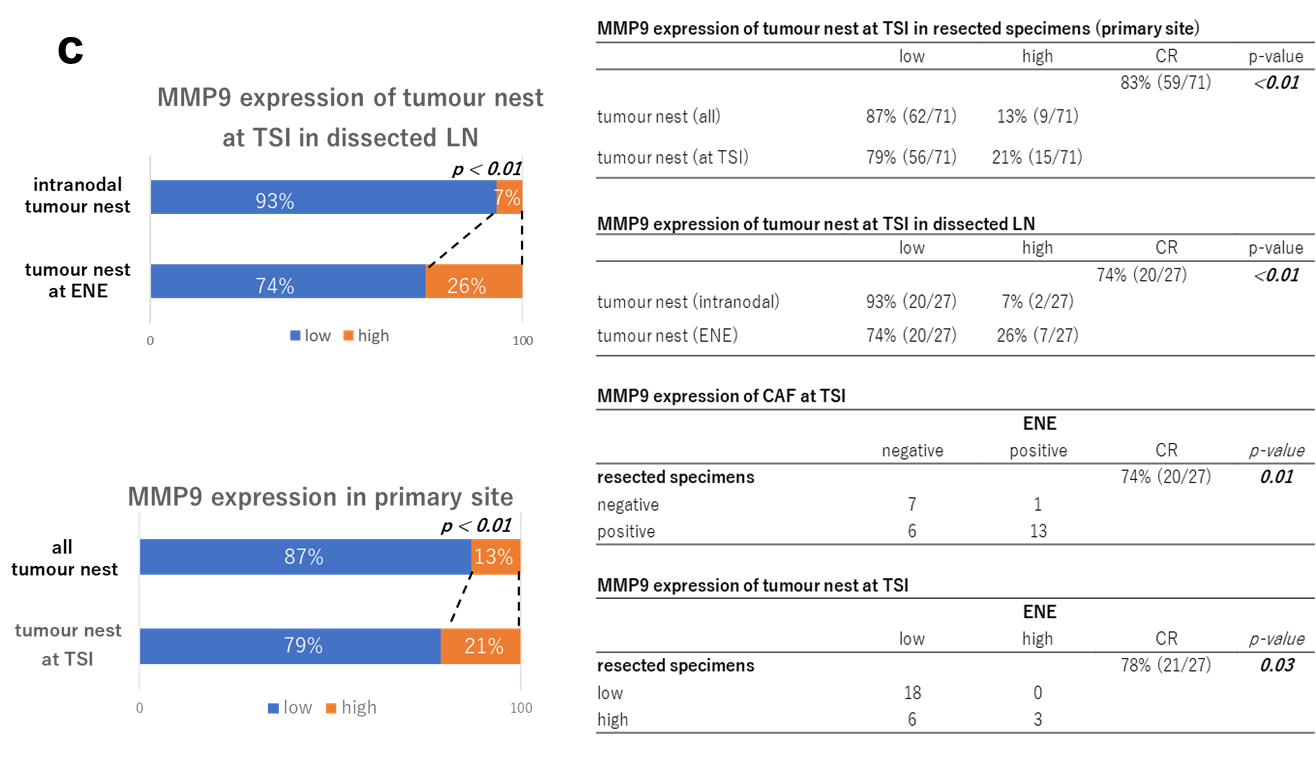


(**a**). MMP2 expression, (**b**). MMP3 expression, (**c**). MMP9 expression.

CAFs, cancer-associated fibroblasts; CR, concordance rate; ENE, extranodal extension; LNs, lymph nodes; TSI: tumour–stromal interface.
