# Supplementary material for: MMP14 expression levels accurately predict the presence of extranodal extensions in oral squamous cell carcinoma: a retrospective cohort study
Source: BMC Cancer. 2023 Feb 10;23:142. doi: 10.1186/s12885-023-10595-x (PMC9921360; doi:10.1186/s12885-023-10595-x)
Supplement: Supplementary file 8 — Supplementary Material 8 [file 12885_2023_10595_MOESM8_ESM.docx]

**Additional File 8.** **Correlation between clinicopathological features and MMP2 expression in the tumour nest and CAFs in 71 OSCC surgically resected specimens**

|  | MMP2 expression in CAFs at the TSI | | | | MMP2 expression in the tumour nest at the TSI | | | |
| --- | --- | --- | --- | --- | --- | --- | --- | --- |
|  | Negative | Positive | Total | *p-*value | Low | High | Total | *p-*value |
| Age |  |  |  | 0.87 |  |  |  | 0.76 |
| > 65 | 12 | 13 | 25 |  | 11 | 14 | 25 |  |
| ≤ 65 | 23 | 23 | 46 |  | 22 | 24 | 46 |  |
| Sex |  |  |  | 0.92 |  |  |  | 0.15 |
| Female | 21 | 22 | 43 |  | 17 | 26 | 43 |  |
| Male | 14 | 14 | 28 |  | 16 | 12 | 28 |  |
| Location |  |  |  | ***0.03*** |  |  |  | 0.44 |
| Buccal mucosa | 3 | 5 | 8 |  | 5 | 3 | 8 |  |
| Gingiva | 5 | 10 | 15 |  | 6 | 9 | 15 |  |
| Tongue | 27 | 21 | 48 |  | 22 | 26 | 48 |  |
| pT |  |  |  | ***0.01*** |  |  |  | ***0.02*** |
| 1.2 | 13 | 4 | 17 |  | 12 | 5 | 17 |  |
| 3.4 | 22 | 32 | 54 |  | 21 | 33 | 54 |  |
| pDOI |  |  |  | 0.06 |  |  |  | ***0.03*** |
| ≤ 10 mm | 14 | 7 | 21 |  | 14 | 7 | 21 |  |
| > 10 mm | 21 | 29 | 50 |  | 19 | 31 | 50 |  |
| Lymph node metastasis | | |  | ***0.01*** |  |  |  | 0.59 |
| (-) | 18 | 8 | 26 |  | 11 | 15 | 26 |  |
| (+) | 17 | 28 | 45 |  | 22 | 23 | 45 |  |
| pN |  |  |  | ***0.01*** |  |  |  | 0.20 |
| 0,1 | 24 | 14 | 38 |  | 15 | 23 | 38 |  |
| 2,3 | 11 | 22 | 33 |  | 18 | 15 | 33 |  |
| ENE |  |  |  | ***0.04*** |  |  |  | 0.09 |
| (-) | 26 | 18 | 44 |  | 17 | 27 | 44 |  |
| (+) | 9 | 18 | 27 |  | 16 | 11 | 27 |  |
| Differentiation | |  |  | 0.72 |  |  |  |  |
| Well | 23 | 22 | 45 |  | 21 | 24 | 45 | 0.44 |
| Moderate | 11 | 13 | 24 |  | 12 | 12 | 24 |  |
| Poor | 1 | 1 | 2 |  | 0 | 2 | 2 |  |
| Invasion pattern | |  |  | 0.48 |  |  |  | 0.42 |
| 1.2 | 4 | 3 | 7 |  | 4 | 3 | 7 |  |
| 3.4c.4d | 31 | 33 | 64 |  | 29 | 35 | 64 |  |
| DR |  |  |  | 0.27 |  |  |  | 0.50 |
| Mature | 13 | 18 | 31 |  | 13 | 18 | 31 |  |
| Immature | 22 | 18 | 40 |  | 20 | 20 | 40 |  |
| TB |  |  |  | 0.24 |  |  |  | 0.06 |
| Low (< 10) | 9 | 14 | 23 |  | 7 | 16 | 23 |  |
| High (≥ 10) | 26 | 22 | 48 |  | 26 | 22 | 48 |  |
| TILs |  |  |  | 0.19 |  |  |  | ***0.01*** |
| High | 18 | 13 | 31 |  | 20 | 11 | 31 |  |
| Low | 17 | 23 | 40 |  | 13 | 27 | 40 |  |
| Ly |  |  |  | ***0.03*** |  |  |  | 0.88 |
| (-) | 14 | 6 | 20 |  | 9 | 11 | 20 |  |
| (+) | 21 | 30 | 51 |  | 24 | 27 | 51 |  |
| V |  |  |  | ***0.04*** |  |  |  | 0.54 |
| (-) | 12 | 5 | 17 |  | 9 | 8 | 17 |  |
| (+) | 23 | 31 | 54 |  | 24 | 30 | 54 |  |
| Pn |  |  |  | 0.55 |  |  |  | 0.69 |
| (-) | 12 | 10 | 22 |  | 11 | 11 | 22 |  |
| (+) | 23 | 26 | 49 |  | 22 | 27 | 49 |  |

CAFs, cancer-associated fibroblasts; TSI, tumour–stromal interface; pT, pathological T; pDOI, pathological depth of invasion; pN, pathological N; ENE, extranodal extension; DR, desmoplastic reaction; OSCC, oral squamous cell carcinoma; TB, tumour budding; TILs, tumour-infiltrating lymphocytes; Ly, lymphatic invasion; V, vascular invasion; Pn, perineural invasion
