# Supplementary material for: MMP14 expression levels accurately predict the presence of extranodal extensions in oral squamous cell carcinoma: a retrospective cohort study
Source: BMC Cancer. 2023 Feb 10;23:142. doi: 10.1186/s12885-023-10595-x (PMC9921360; doi:10.1186/s12885-023-10595-x)
Supplement: Supplementary file 10 — Supplementary Material 10 [file 12885_2023_10595_MOESM10_ESM.docx]

**Additional File 10. Correlation between MMP3 expression and clinicopathological features of the tumour nest and CAFs in OSCC resection specimens**

|  | MMP3 expression in CAFs  at the TSI | | | | MMP3 expression in the tumour nest at the TSI | | | |
| --- | --- | --- | --- | --- | --- | --- | --- | --- |
|  | Negative | Positive | Total | *p-*value | Low | High | Total | *p-*value |
| Age |  |  |  | 0.38 |  |  |  | 0.60 |
| > 65 | 16 | 9 | 25 |  | 17 | 8 | 25 |  |
| ≤ 65 | 34 | 12 | 46 |  | 34 | 12 | 46 |  |
| Sex |  |  |  | 0.36 |  |  |  | 0.25 |
| Female | 32 | 11 | 43 |  | 33 | 10 | 43 |  |
| Male | 18 | 10 | 28 |  | 18 | 10 | 28 |  |
| Location |  |  |  | 0.06 |  |  |  | 0.61 |
| Buccal mucosa | 8 | 0 | 8 |  | 6 | 2 | 8 |  |
| Gingiva | 12 | 3 | 15 |  | 11 | 4 | 15 |  |
| Tongue | 30 | 18 | 48 |  | 34 | 14 | 48 |  |
| pT |  |  |  | 0.07 |  |  |  | 0.90 |
| 1.2 | 15 | 2 | 17 |  | 12 | 5 | 17 |  |
| 3.4 | 35 | 19 | 54 |  | 39 | 15 | 54 |  |
| pDOI |  |  |  | 0.057 |  |  |  | 0.23 |
| ≤ 10 mm | 18 | 3 | 21 |  | 13 | 8 | 21 |  |
| > 10 mm | 32 | 18 | 50 |  | 38 | 12 | 50 |  |
| Lymph node metastasis | | |  | 0.14 |  |  |  | 0.47 |
| (-) | 21 | 5 | 26 |  | 20 | 6 | 26 |  |
| (+) | 29 | 16 | 45 |  | 31 | 14 | 45 |  |
| pN |  |  |  | 0.52 |  |  |  | 0.88 |
| 0,1 | 28 | 10 | 38 |  | 27 | 11 | 38 |  |
| 2,3 | 22 | 11 | 33 |  | 24 | 9 | 33 |  |
| ENE |  |  |  | 0.59 |  |  |  | 0.83 |
| (-) | 32 | 12 | 44 |  | 32 | 12 | 44 |  |
| (+) | 18 | 9 | 27 |  | 19 | 8 | 27 |  |
| Differentiation | |  |  | 0.58 |  |  |  | 0.58 |
| Well | 33 | 12 | 45 |  | 33 | 12 | 45 |  |
| Moderate | 15 | 9 | 24 |  | 17 | 7 | 24 |  |
| Poor | 2 | 0 | 2 |  | 1 | 1 | 2 |  |
| Invasion pattern | |  |  | 0.33 |  |  |  | 0.69 |
| 1.2 | 6 | 1 | 7 |  | 5 | 2 | 7 |  |
| 3.4c.4d | 44 | 20 | 64 |  | 46 | 18 | 64 |  |
| DR |  |  |  | 0.31 |  |  |  | 0.40 |
| Mature | 28 | 9 | 37 |  | 25 | 12 | 37 |  |
| Immature | 22 | 12 | 34 |  | 26 | 8 | 34 |  |
| TB |  |  |  | 0.26 |  |  |  | 0.50 |
| Low (< 10) | 24 | 7 | 31 |  | 21 | 10 | 31 |  |
| High (≥ 10) | 26 | 14 | 40 |  | 30 | 10 | 40 |  |
| TILs |  |  |  | 0.26 |  |  |  | 0.89 |
| High | 24 | 7 | 31 |  | 22 | 9 | 31 |  |
| Low | 26 | 14 | 40 |  | 29 | 11 | 40 |  |
| Ly |  |  |  | 0.21 |  |  |  | 0.26 |
| (-) | 16 | 4 | 20 |  | 16 | 4 | 20 |  |
| (+) | 34 | 17 | 51 |  | 35 | 16 | 51 |  |
| V |  |  |  | ***0.01*** |  |  |  | 0.44 |
| (-) | 16 | 1 | 17 |  | 13 | 4 | 17 |  |
| (+) | 34 | 20 | 54 |  | 38 | 16 | 54 |  |
| Pn |  |  |  | ***0.01*** |  |  |  | 0.91 |
| (-) | 20 | 2 | 22 |  | 16 | 6 | 22 |  |
| (+) | 30 | 19 | 49 |  | 35 | 14 | 49 |  |

CAFs, cancer-associated fibroblasts; TSI, tumour–stromal interface; pT, pathological T; pDOI, pathological depth of invasion; pN, pathological N; ENE, extranodal extension; DR, desmoplastic reaction; OSCC, oral squamous cell carcinoma; TB, tumour budding; TILs, tumour-infiltrating lymphocytes; Ly, lymphatic invasion; V, vascular invasion; Pn, perineural invasion
