# Supplementary material for: MMP14 expression levels accurately predict the presence of extranodal extensions in oral squamous cell carcinoma: a retrospective cohort study
Source: BMC Cancer. 2023 Feb 10;23:142. doi: 10.1186/s12885-023-10595-x (PMC9921360; doi:10.1186/s12885-023-10595-x)
Supplement: Supplementary file 11 — Supplementary Material 11 [file 12885_2023_10595_MOESM11_ESM.docx]

**Additional File 11.** **Correlation between MMP14 expression in all tumour nests, and total MMP14 expression in CAFs at the TSI and the tumour nest with clinicopathological features of the OSCC resections**

|  | MMP14 expression in  all tumour nests | | | | Total MMP14 expression in the CAFs at the TSI and all tumour nests | | | |
| --- | --- | --- | --- | --- | --- | --- | --- | --- |
|  | Negative | Positive | Total | *p-*value | Low-risk | High-risk | Total | *p-*value |
| Age |  |  |  | 0.86 |  |  |  | 0.84 |
| > 65 | 20 | 5 | 25 |  | 21 | 4 | 25 |  |
| ≤ 65 | 36 | 10 | 46 |  | 41 | 5 | 46 |  |
| Sex |  |  |  | 0.59 |  |  |  | 0.49 |
| Female | 33 | 10 | 43 |  | 37 | 6 | 43 |  |
| Male | 23 | 5 | 28 |  | 25 | 3 | 28 |  |
| Location |  |  |  | 0.61 |  |  |  | 0.61 |
| Buccal mucosa | 7 | 1 | 8 |  | 8 | 0 | 8 |  |
| Gingiva | 13 | 2 | 15 |  | 14 | 1 | 15 |  |
| Tongue | 36 | 12 | 48 |  | 40 | 8 | 48 |  |
| pT |  |  |  | 0.23 |  |  |  | 0.07 |
| 1.2 | 15 | 2 | 17 |  | 17 | 0 | 17 |  |
| 3.4 | 41 | 13 | 54 |  | 45 | 9 | 54 |  |
| pDOI |  |  |  | 0.28 |  |  |  | 0.19 |
| ≤ 10 mm | 18 | 3 | 21 |  | 20 | 1 | 21 |  |
| > 10 mm | 38 | 12 | 50 |  | 42 | 8 | 50 |  |
| Lymph node metastasis | | |  | 0.11 |  | |  | ***0.01*** |
| (-) | 23 | 3 | 26 |  | 26 | 0 | 26 |  |
| (+) | 33 | 12 | 45 |  | 36 | 9 | 45 |  |
| pN |  |  |  | 0.55 |  |  |  | 0.41 |
| 0,1 | 31 | 7 | 38 |  | 34 | 4 | 38 |  |
| 2,3 | 25 | 8 | 33 |  | 28 | 5 | 33 |  |
| ENE |  |  |  | 0.44 |  |  |  | 0.21 |
| (-) | 36 | 8 | 44 |  | 40 | 4 | 44 |  |
| (+) | 20 | 7 | 27 |  | 22 | 5 | 27 |  |
| Differentiation | |  |  | 0.14 |  |  |  | 0.71 |
| Well | 38 | 7 | 45 |  | 39 | 6 | 45 |  |
| Moderate | 18 | 6 | 24 |  | 21 | 3 | 24 |  |
| Poor | 0 | 2 | 2 |  | 2 | 0 | 2 |  |
| Invasion pattern | |  |  | 0.17 |  |  |  | 0.37 |
| 1.2 | 7 | 0 | 7 |  | 7 | 0 | 7 |  |
| 3.4c.4d | 49 | 15 | 64 |  | 55 | 9 | 64 |  |
| DR |  |  |  | 0.22 |  |  |  | 0.44 |
| Mature | 31 | 6 | 37 |  | 33 | 4 | 37 |  |
| Immature | 25 | 9 | 34 |  | 29 | 5 | 34 |  |
| TB |  |  |  | ***0.03*** |  |  |  | ***0.04*** |
| Low (< 10) | 28 | 3 | 31 |  | 30 | 1 | 31 |  |
| High (≥ 10) | 28 | 12 | 40 |  | 32 | 8 | 40 |  |
| TILs |  |  |  | 0.75 |  |  |  | 0.38 |
| High | 25 | 6 | 31 |  | 28 | 3 | 31 |  |
| Low | 31 | 9 | 40 |  | 34 | 6 | 40 |  |
| Ly |  |  |  | ***0.03*** |  |  |  | ***0.04*** |
| (-) | 19 | 1 | 20 |  | 20 | 0 | 20 |  |
| (+) | 37 | 14 | 51 |  | 42 | 9 | 51 |  |
| V |  |  |  | 0.07 |  |  |  | 0.07 |
| (-) | 16 | 1 | 17 |  | 17 | 0 | 17 |  |
| (+) | 40 | 14 | 54 |  | 45 | 9 | 54 |  |
| Pn |  |  |  | 1.30 |  |  |  | 0.43 |
| (-) | 18 | 4 | 22 |  | 20 | 2 | 22 |  |
| (+) | 38 | 11 | 49 |  | 42 | 7 | 49 |  |

CAFs, cancer-associated fibroblasts; TSI, tumour–stromal interface; pT, pathological T; pDOI, pathological depth of invasion; pN, pathological N; ENE, extranodal extension; DR, desmoplastic reaction; OSCC, oral squamous cell carcinoma; TB, tumour budding; TILs, tumour-infiltrating lymphocytes; Ly, lymphatic invasion; V, vascular invasion; Pn, perineural invasion
