# Supplementary material for: MMP14 expression levels accurately predict the presence of extranodal extensions in oral squamous cell carcinoma: a retrospective cohort study
Source: BMC Cancer. 2023 Feb 10;23:142. doi: 10.1186/s12885-023-10595-x (PMC9921360; doi:10.1186/s12885-023-10595-x)
Supplement: Supplementary file 12 — Supplementary Material 12 [file 12885_2023_10595_MOESM12_ESM.docx]

**Additional File 12. Concordance rate (CR) of MMP14 expression among biopsies, ENE sites, and resected specimens**


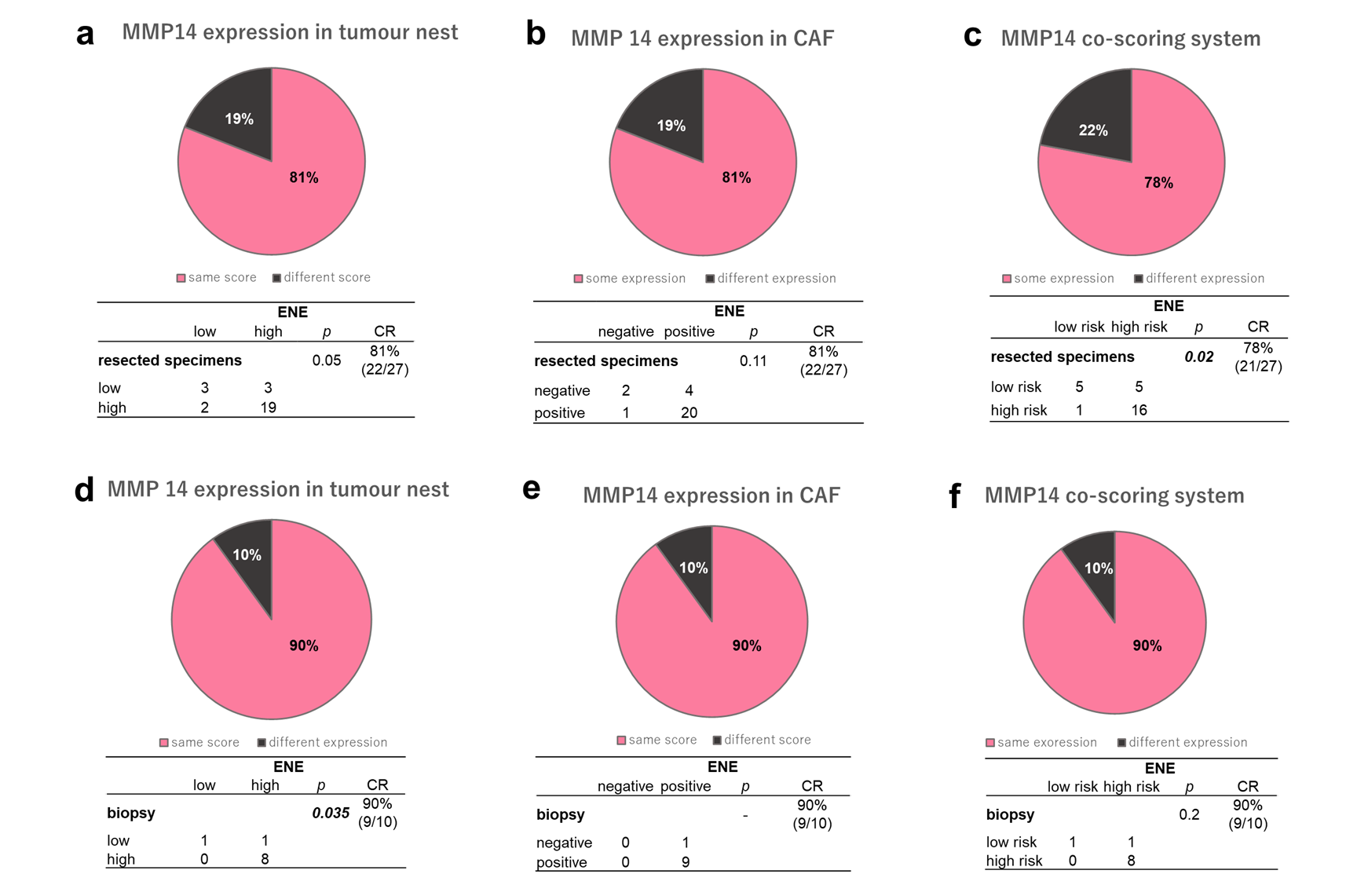


A high CR was found at the tumour nest (**a**, 81%), in cancer-associated fibroblasts (CAFs) (**b**, 81%) and in the total score group (**c**, 78%) between extranodal extension (ENE) sites and surgically resected specimens. Similar results were found between ENE sites and biopsy specimens in the MMP14 co-scoring system (**d−f**), at the tumour nest (**d**, 90%), in CAFs (**e**, 90%), and the MMP14 co-scoring system (**f**, 90%).
